# Bridging Atomistic and Mesoscale Lithium Transport via Machine-Learned Force Fields and Markov State Models

**DOI:** 10.1021/acs.jctc.5c02035

**Published:** 2026-05-20

**Authors:** Muhammad Nawaz Qaisrani, Christoph Kirsch, Aaron Flötotto, Jonas Hänseroth, Jules Jean Max Oumard, Daniel Sebastiani, Christian Dreßler

**Affiliations:** † 26559 Ilmenau University of Technology, Theoretical Solid State Physics, Weimarer Straße 32, 98693 Ilmenau, Germany; ‡ 229897 Martin-Luther-University Halle-Wittenberg, Institute of Chemistry, Theoretical Chemistry, Von-Danckelmann-Platz 4, 06120 Halle (Saale), Germany

## Abstract

Lithium diffusion in silicon battery anodes is governed by thermally activated jumps between (meta)­stable sites separated by significant energy barriers, making such events rare on *ab initio* molecular dynamics (AIMD) time scales. To overcome this limitation, we establish a multiscale workflow that links AIMD, machine-learned force fields (MLFFs), and Markov state models (MSMs) to bridge atomistic mechanisms to mesoscale diffusion. Focusing on crystalline Li–Si phases, our MLFFs trained on AIMD data, achieve near-DFT accuracy while enabling large-scale molecular dynamics simulations extending to tens of nanoseconds. From these trajectories, we extract converged lithium-jump statistics to construct MSMs that quantitatively reproduce diffusivities with uncertainties an order of magnitude smaller than those obtained from 100 ps AIMD simulations. Demonstrated here for crystalline Li_*x*_Si_*y*_ phases, the AIMD → MLFF → MSM workflow provides a transferable route for quantitative transport modeling in amorphous structures, defect-mediated diffusion, and alternative solid-state anodes.

## Graphical abstract

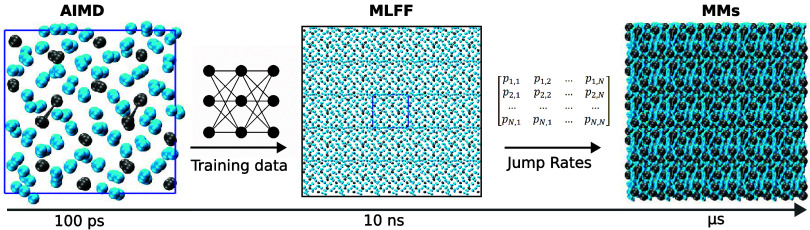

## 1 Introduction

Lithium-ion batteries power technologies from portable electronics to electric vehicles and grid-scale storage, yet their performance and lifetime are tightly coupled to ion transport within electrode materials.^1–4^ In particular, the anode critically influences the achievable energy density, charging kinetics, and mechanical stability of the cell.^5–8^ Graphite, the dominant commercial anode, provides reliable cycling but is constrained by a modest capacity of 372 mAh/g, motivating the search for higher-capacity alternatives.^9–11^ Silicon stands out as a promising candidate with a theoretical capacity of 3579 mAh/g in its fully lithiated Li_15_Si_4_ state, nearly ten times that of graphite.^12,13^

Despite this advantage, silicon anodes face severe challenges. Upon lithiation, they undergo volume changes of up to 380%, leading to mechanical degradation and rapid capacity fading.^13–21^ Understanding lithium transport is central to addressing these issues, as diffusion kinetics govern charge rates, stress evolution, and overall electrochemical stability. While amorphous silicon dominates in practical electrodes, its structural disorder complicates systematic analysis. Crystalline lithium silicides (Li_*x*_Si_*y*_), by contrast, provide well-defined diffusion pathways and serve as controlled model systems for probing lithium mobility and benchmarking computational methods.^22–24^ Insights obtained from such ordered phases can be transferred to more complex amorphous systems, offering a clean and interpretable starting point for multiscale transport modeling.

Recent progress in machine learning has transformed our ability to model lithium diffusion at the atomic scale. Machine-learned force fields (MLFFs) have emerged as an efficient solution to the traditional accuracy–efficiency trade-off. By training directly on quantum-mechanical reference data, MLFFs can reach near-DFT accuracy while enabling nanosecond-scale simulations in large supercells. Frameworks such as the Behler–Parrinello neural network potentials,^25^ Gaussian approximation potentials (GAP),^26^ and modern equivariant graph neural networks like NequIP^27^ and MACE^28^ have demonstrated remarkable versatility in capturing chemically diverse bonding environments. In the context of batteries, MLFFs have been successfully applied to lithium dynamics in metal surfaces,^29^ amorphous electrodes,^30^ graphite,^31^ and solid electrolytes such as Li_3_TiCl_6_,^32^ highlighting their potential for a broad range of electrochemical systems.

The development of reactive and machine-learned force fields for lithium diffusion in silicon anodes is an active area of research.^33–37^ Although these approaches dramatically extend the accessible time and length scales compared to *ab initio* molecular dynamics, they remain fundamentally atomistic. Yet, real anodes exhibit mesoscale complexity—nanostructured morphologies, heterogeneous lithium concentrations, and evolving microstructures designed to accommodate large volume expansion during lithiation.^16–21^ In such environments, lithium motion involves collective and correlated ion dynamics spanning microseconds and micrometers. Bridging this gap between atomic detail and mesoscale transport requires systematic coarse-graining strategies that preserve the underlying physics of atomistic motion.

A particularly powerful strategy is to extract lithium jump statistics from long MLFF trajectories and represent them using Markov state models (MSMs).^38–40^ MSMs recast ion motion as probabilistic transitions between (meta)­stable sites, providing a rigorous statistical framework for coarse-graining atomistic dynamics and extrapolating diffusion behavior over extended temporal and spatial scales. In this representation, the long-time scale evolution of the system is captured by the repeated application of a transition matrix, allowing atomistic dynamics to be projected onto a coarse-grained, stochastic model. A key advantage of MSMs is that they make otherwise inaccessible time scales tractable through efficient propagation of state probabilities, i.e., several picoseconds of system dynamics can be obtained through simple matrix–vector multiplications.

The reliability of an MSM depends critically on two factors: the choice of lag time and the quality of sampling. The lag time must be long enough to average out rapid local fluctuations, ensuring Markovian behavior, but also short enough to resolve the relevant kinetic processes; if chosen too large, distinct transition pathways may be lumped together and important dynamical information lost.

At the same time, sufficient statistics are needed to estimate transition probabilities with confidence. These conditions are particularly challenging for *ab initio* molecular dynamics, where accessible trajectories are typically short. Validation procedures such as the Chapman–Kolmogorov test and the analysis of implied time scales provide practical means to assess whether an MSM is consistent and predictive.

In this work, we integrate these three components into a unified bottom-up workflow, (AIMD → MLFF → MSM), that links quantum-accurate barriers to mesoscale transport. AIMD supplies high-fidelity reference data for model training, MLFFs extend simulation times and system sizes to capture rare hopping events, and MSMs coarse-grain the resulting trajectories into statistically consistent transport models. We demonstrate this approach for crystalline Li–Si phases as a controlled test case, establishing a quantitative and transferable foundation for modeling lithium transport in amorphous and defect-rich silicon anodes relevant to next-generation battery technologies.

## 2 Theory

### 2.1 Markov State Models (MSM)

Markov state models (MSMs) provide a compact kinetic description of complex molecular systems by representing their dynamics as transitions between a finite number of discrete states. They are attractive because the temporal evolution of the system can be propagated efficiently through repeated multiplication with a transition matrix, allowing access to long time scales. At the same time, MSMs offer a mathematically controlled analogue of the human process of identifying characteristic configurations and transitions in a trajectory. Whereas visual inspection of a simulation often leads to qualitative and potentially biased “look-and-see” interpretations, MSMs provide an objective criterion for assessing whether the inferred transition network represents a meaningful dynamical model.

The construction of an MSM begins by discretizing the continuous phase space into a finite set of states. Transition probabilities between these states over a lag time τ are then estimated (e.g., from MD trajectories), yielding a transition matrix $M^{\tau }$ that propagates the probability vector according to

1 $$x^{t+\tau }=M^{\tau }x^{t}$$

To quantitatively assess whether a transition matrix satisfies the Markov property, one can employ the Chapman–Kolmogorov (CK) test, which is central to MSM theory. The CK test (eq 2) is the rigorous quantitative validation of the dynamic model, which is missing in “look and see” experiments. The CK test evaluates whether the transition matrix estimated at lag time τ contains sufficient information to predict the dynamics over longer times *n*τ. Formally, a Markovian model must satisfy

2 $${\left(M_{\mathrm{sampled}}^{\tau }\right)}^{n}\approx M_{\mathrm{sampled}}^{n\tau }$$

This condition is often interpreted merely as a technical check for Markovianity. However, its conceptual significance is deeper. It tests whether the short-lag transition matrix $M^{\tau }$ contains all dynamical information required to predict the future evolution of the system. If the CK test holds, the dynamics on the interval *n*τ can be reconstructed from repeated application of the single-step matrix, implying that $M^{\tau }$ provides a complete and self-consistent description of the kinetics. When the CK test fails, the MSM can not reliably reproduce the underlying dynamics for this discretization of the state space.

For equilibrium simulations it is important to note that for sufficiently large lag times every transition matrix becomes trivial, because all states relax to the stationary distribution. In this limit each column of the transition matrix approaches the equilibrium distribution. Such matrices automatically satisfy the CK test but contain no microscopically resolved kinetic information. We refer to these cases as *nonmeaningful MSMs*, since they describe only the equilibrium distribution rather than the dynamical pathways leading to it.

The discretization of the phase space is a central ingredient in the construction of any MSM. When derived from MD simulations, this discretization typically involves a drastic reduction of dimensionality. Such coarse-graining inevitably introduces memory effects, implying that the resulting dynamics become approximately Markovian only beyond a minimal lag time τ. Meaningful MSMs can only be constructed if the chosen discretization reflects the physical nature of the underlying dynamical process. In that case the transition matrix becomes Markovian at lag times where relevant dynamical processes are still resolved and have not decayed.

In contrast, a nonmeaningful MSM may also satisfy the Markov property, but only at a lag time τ_0_ so large that the corresponding transition matrix $M^{\tau_{0}}$ already contains the stationary distribution in each column.

These ideas are illustrated by the instructive toy model shown in Figure 1a, which consists of six particles hopping among 12 lattice sites with periodic boundary conditions. The two investigated variants of the dynamics are described as site-to-site transitions in Figure 1a,1b. In the first case (a), the particles move independently and lattice sites may be occupied by multiple particles. In the second case (b), the particles exhibit a weak form of correlation: each lattice site may be occupied by at most one particle. Importantly, the hopping probabilities themselves are not affected by the presence of neighboring particles. Using the lattice sites as discrete states, transition matrices are estimated from trajectories generated for this model system.

**Figure 1:**
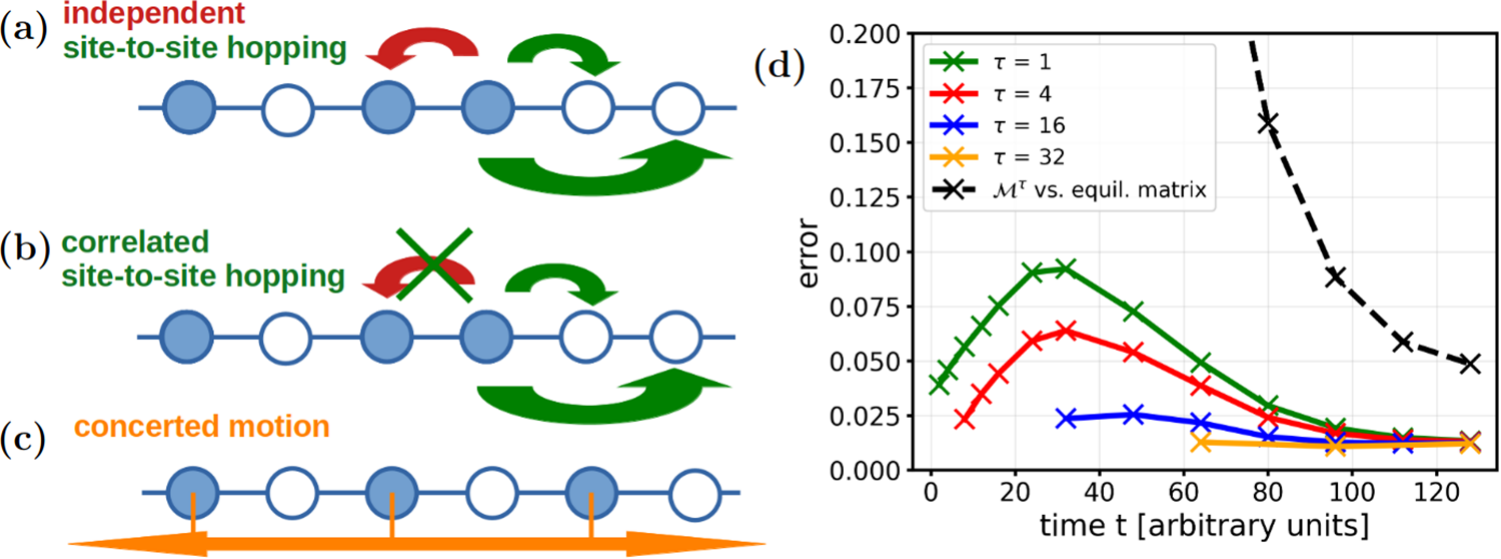
Instructive toy model illustrating the limitations of Markov state models constructed on small state spaces for correlated dynamics. (a–c) Possible jump mechanisms on a one-dimensional lattice: (a) independent site-to-site hopping, (b) correlated site-to-site hopping by restricting each lattice site to single occupancy and (c) concerted motion (all particles move simultaneously). Please note that for the 1D case, independent particle motion and concerted motion cannot be distinguished by a MSM using lattice sites as state space and lead to identical transition matrices. (d) Chapman–Kolmogorov (CK) test for the correlated case (shown in (b)) using lattice sites as the MSM state space. The error err­(*n*) (eq 6) compares the sampled multilag transition matrix $M_{\mathrm{sampled}}^{n\tau }$ with the Markov prediction ${\left(M^{\tau }\right)}^{n}$ . The dashed black line indicates the deviation from the stationary-distribution matrix. Because the CK condition is satisfied only at lag times where the system has already reached the stationary distribution, only a trivial MSM can be obtained.

For the independent system (Figure 1a), the 12-state MSM satisfies the Chapman–Kolmogorov (CK) test without requiring a finite lag time τ, because the discretization retains the full dimensionality of the underlying dynamics. In contrast, the correlated system (Figure 1b) violates the CK test up to large lag times. When the test is finally satisfied, the transition matrix is already nearly identical to a matrix whose columns correspond to the stationary distribution (dashed black line in Figure 1d). In this situation only a trivial MSM can be constructed. This example demonstrates that even very weak correlations can introduce sufficient memory to prevent the construction of nontrivial MSMs.

The natural question is therefore what is the dimension of the state space for which a transition matrix would satisfy the CK test without requiring a lag time? To answer this question we must shift the perspective from transitions between single-particle lattice sites to *N*-body configurations of the particles. In this representation the elementary states correspond to all possible arrangements of *N* particles among *Ñ* lattice sites. The number of such configurations is $\left(\begin{array}{l} Ñ\\ N \end{array}\right)$ . Consequently, the true state space for correlated dynamics becomes combinatorially large and the construction of MSMs quickly becomes infeasible, even for moderate system sizes, because sampling the corresponding transition matrices becomes numerically intractable. For the toy model of six particles hopping among 12 lattice sites, the dimension of the true dynamical state space is $\left(\begin{array}{l} 12\\ 6 \end{array}\right)$ .

For the toy model we considered correlations only among the mobile particles. For ionic transport in general, and in particular for Li_*x*_Si_*y*_, several mechanisms are possible. Ion transport may involve (i) independent ion motion (independent Li jumps), (ii) correlated motion among mobile ions (Li motion correlated with other Li ions), or (iii) correlated motion involving both mobile ions and host atoms (Li motion correlated with Li and Si atoms).

From the instructive toy example it is clear that the dimension of the state space describing the true dynamics increases drastically from case (i) to case (iii), since correlations involve an increasing number of particles. The number of possible states is determined by all possible distributions of the relevant particles among the available lattice sites. Table S3 in the SI summarizes the corresponding state-space sizes and the minimal lag time τ_0_ required for the transition matrix to satisfy the CK test for cases (i)–(iii). Coarse-graining the dynamics inevitably introduces memory effects, such that the model becomes Markovian only at lag times exceeding a single timestep (τ_0_ > 1).

Following this discussion, it appears unlikely that ionic motion in one-dimensional channels can generally be described by a simple site-to-site model that uses only the crystallographic lattice sites as the discretization of the state space. Such a model would correspond to independent ion motion in an effective bath formed by the other mobile ions.

In this work we demonstrate, however, that for moderate lag times it is possible to construct meaningful Markov models for crystalline Li_*x*_Si_*y*_ phases using only the small state space defined by the crystallographic lattice sites. Although ionic motion is never purely independent due to Coulomb interactions between the ions, the MSM analysis allows us to understand how these correlations are effectively incorporated into the coarse-grained kinetic model. Finally, the spectral properties of the transition matrix can provide mechanistic insight. The leading eigenvalue λ_1_ = 1 corresponds to the stationary distribution, while all other eigenvalues |λ_*k*_| < 1 describe relaxation modes. Their associated implied time scales

$$t_{k}\left(\tau \right)=-\frac{\tau }{\ln|\lambda_{k}\left(\tau )\right|}$$ 3

are expected to form plateaus as a function of τ when a consistent MSM exists. The absence of such plateaus, as an alternative to direct inspection of the transition matrix $M^{\tau }$ , provides clear evidence that only trivial MSMs can be constructed.

### 2.2 Central Idea of the AIMD → MLFF → MSM Approach

This work integrates *ab initio* molecular dynamics (AIMD), machine-learned force fields (MLFFs), and Markov state models (MSMs) into a unified multiscale framework. AIMD was employed to generate reference data sets for training and validating MLFFs. The fine-tuned MLFFs then enabled nanosecond-scale molecular dynamics trajectories, from which lithium jump statistics were extracted. These statistics were subsequently coarse-grained into MSMs, providing a stochastic representation of ion transport over extended spatial and temporal scales. The following sections detail the simulation protocols and model construction steps.

#### 2.2.1 *Ab Initio* Molecular Dynamics Simulations

The *ab initio* molecular dynamics trajectories used in this study were taken from our previously published work,^41^ in which defect-free Li_12_Si_7_ and Li_13_Si_4_ systems were simulated using the CP2K package.^42^ Simulations were performed in the NVT ensemble using the Becke–Lee–Yang–Parr (BLYP) exchange–correlation functional,^43,44^ GTH pseudopotentials,^45^ and DZVP-MOLOPT-SR-GTH basis sets.^46^ Dispersion interactions were included via the DFT-D3 method,^47^ and a time step of 0.5 fs was employed with a Nosé–Hoover chain thermostat.^48^ Both systems were equilibrated and simulated at 500 K producing 100 ps of trajectory. From these trajectories, 200 or 2000 frames per system were extracted to construct training data sets, ensuring diverse coverage of local atomic environments.

#### 2.2.2 Fine-Tuning of MLFFs

Machine-learned force fields were developed within the MACE framework,^28^ implemented via the MACE Python package (v0.3.10). We initialized from the publicly available MACE-MP-0 foundation model and fine-tuned it on system-specific DFT reference data extracted from AIMD trajectories.^49,50^ For each system, two fine-tuned models were trained: one on a reduced data set (200 frames) and one on an extended data set (2000 frames). A detailed explanation of the finetuning protocol is given in the SI. The fine-tuning protocol was carried out using the workflow implemented in the aMACEing_toolkit package.^51^

#### 2.2.3 MLFF Molecular Dynamics Simulations

Molecular dynamics simulations were performed using the fine-tuned MACE models interfaced through the Atomic Simulation Environment (ASE).^52^ All simulations were carried out in the NVT ensemble with a Langevin thermostat to maintain a constant temperature of 500 K. To validate the results, equivalent simulations were repeated with the LAMMPS package using a Nosé–Hoover chain thermostat.^53^ An integration time step of 0.5 fs was used throughout. Trajectories for 10–30 ns were generated for the Li_*x*_Si_*y*_ systems.

These extended time scales were crucial for capturing rare hopping events and collective ionic motion, and for achieving statistically converged transport properties. Periodic boundary conditions were applied in all three spatial directions to minimize finite-size effects. The molecular dynamics simulations were prepared through the workflow provided by the aMACEing_toolkit package.^51^

#### 2.2.4 Markov Model Construction and Convergence

The construction of Markov State Models (MSMs) from molecular dynamics (MD) trajectories proceeds in four steps:

**Step 1: Discretization of phase space**: Lithium transport in the Li_*x*_Si_*y*_ compound class can be described as a sequence of hops between lattice sites. Here, we use the crystallographic Li positions as lattice sites. Since each of these sites is initially occupied by one Li atom, the number of lattice sites equals the number of Li atoms *N*. If the simulated system contains *N* lithium atoms, the system is represented by an *N*-dimensional state vector *x⃗*, where the *i*th component of *x⃗* corresponds to the fraction of lithium atoms residing at the *i*th lattice site.

**Step 2: Sampling of the transition matrix**: Let S={1,...,N} denote the set of sites and s(t)∈S the site occupied at time *t*. Sampling at a lag time τ yields pairs (*s*(*t*), *s*(*t* + τ)), from which the count matrix is obtained as

4 $$C_{\mathrm{ij}}\left(\tau \right)=\#\left\{t:s\left(t\right)=j,s\left(t+\tau \right)=i\right\}$$

together with the corresponding column-stochastic transition matrix

5 $$M_{\mathrm{ij}}^{\tau }=\frac{C_{\mathrm{ij}}\left(\tau \right)}{\sum_{i}C_{\mathrm{ij}}\left(\tau \right)},,\sum_{i}M_{\mathrm{ij}}^{\tau }=1$$

**Step 3: Validation of Markovianity**: The validity of the Markov assumption was assessed through (i) the analysis of implied time scales and (ii) the Chapman–Kolmogorov (CK) test.

The plateau region of the implied time scales (eq 3) defines the minimal lag time τ_min_ for each eigenvalue. For practical applications, we focus only on the implied time scales corresponding to those eigenstates that contribute most significantly to the relaxation dynamics toward equilibrium. Selecting τ from the plateau region of these implied time scales ensures that the resulting MSM remains both Markovian and predictive.

The CK test [eq 2] evaluates the consistency of the Markov property by comparing transition matrices sampled at multiples of a fixed lag time τ with powers of the single-lag matrix

6 $$\mathrm{err}\left[n\right]=\frac{∥M_{\mathrm{sampled}}^{n\tau }-(M^{\tau }{)}^{n}{∥}_{2}}{∥M_{\mathrm{sampled}}^{n\tau }{∥}_{2}}$$

where ∥·∥_2_ denotes the matrix 2-norm and *n* the length of the Markov chain.

**Step 4: Calculation of the Mean-Square Displacement (MSD)**: Transport properties are reconstructed by propagating displacements through the MSM. The matrix element ${\left(M^{n\tau }\right)}_{\mathrm{ij}}$ gives the probability of transfer from site *j* to site *i* after *n* steps. Denoting the distance between sites *i* and *j* as *d*_*ij*_, the mean-square displacement (MSD) is given by

7 $$\mathrm{MSD}\left(n\tau \right)=\sum_{\mathrm{ij}}{\left(M^{n\tau }\right)}_{\mathrm{ij}}d_{\mathrm{ij}}^{2}$$

The diffusivity *D* is then obtained from the long-time slope of the MSD using the Einstein relation

8 $$D=\lim_{t\to \infty }\frac{1}{2d}\frac{d}{dt}\left\langle \right|\Delta r\left(t\right){|}^{2}\rangle ,,d=3$$

Because ion propagation via the transition matrix does not account for periodic boundary crossings, diffusion is effectively confined to the dimensions of the MD simulation cell. As a result, the MSD derived from transition matrices exhibits a plateau at very long time scales. To mitigate this artifact, we construct transition matrices for replicated supercells, thereby shifting the plateau to time scales beyond 1 ns and recovering the diffusive regime within the accessible temporal window.

## 3 Results

We began by assessing the accuracy of the machine-learned force fields (MLFFs) trained within the MACE framework. Training data sets were generated for Li_12_Si_7_ and Li_13_Si_4_, and training performance was evaluated using root-mean-square errors (RMSE) in total energies and atomic forces (Table S1). Across both phases, the fine-tuned models achieved energy errors below 2 meV atom^–1^ and force errors below 30 meV Å^–1^. Additional validation against direct DFT evaluations along independent MLFF-driven MD trajectories (Table S2) showed comparable deviations in forces and energies, suggesting that the models can reproduce short-time dynamical behavior such as lithium hopping. The resulting MLFFs therefore provide a reliable description of the potential-energy surfaces for extended simulations of lithium transport in Li_*x*_Si_*y*_ compounds. Previous studies have also reported machine-learned force fields for lithium silicides.^33–35^ These models, typically based on deep neural network architectures, reported force RMSEs on the order of 100 meV Å^–1^. In comparison, our fine-tuned MACE models achieve lower errors with similar training-set sizes, reflecting the advantages of equivariant graph neural networks that preserve rotational symmetries and incorporate angular and many-body correlations in local atomic environments. These features contribute to improved data efficiency and a more accurate representation of local structural interactions within the tested systems.

We next examined whether the fine-tuned models also reproduce structural correlations characteristic of the underlying phases. Figure 2 compares radial distribution functions (RDFs) of Li_12_Si_7_ at 500 K obtained from AIMD, the pretrained MACE foundation model, and the fine-tuned MLFF. While the foundation model captures short-range features of *g*(*r*), systematic deviations appear at intermediate and long distances, particularly for Si–Si correlations, reflecting the challenge of describing diverse bonding motifs in Li–Si networks with a general-purpose model. Fine-tuning substantially improves agreement across all pair types: the positions and amplitudes of Li–Si and Si–Si peaks are accurately reproduced, and the long-range decay of *g*(*r*) closely follows AIMD. The Li–Li peak amplitude is slightly overestimated, but the overall structural ordering remains consistent with DFT-based reference data. A similar level of agreement is observed for Li_13_Si_4_ (Figure S1), confirming that the fine-tuned MLFFs reliably capture both local and extended order across distinct crystalline Li–Si compositions.

**Figure 2:**
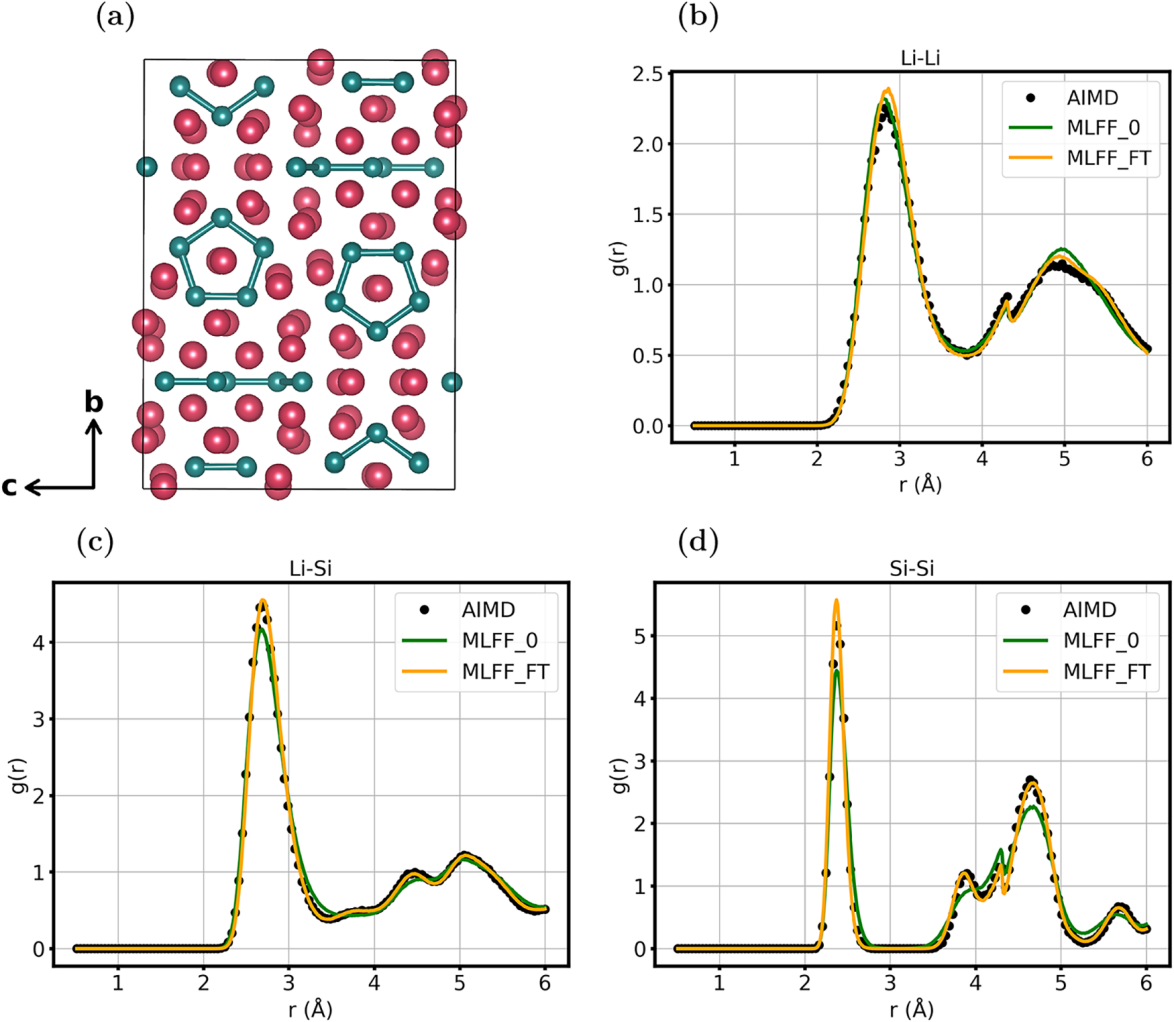
(a) Snapshot of the Li_12_Si_7_ crystal structure. Lithium and silicon atoms are shown in red and teal, respectively. Panels (b–d) illustrate comparisons of the radial distribution function *g*(*r*) obtained from AIMD (black filled circles), the MACE foundation model (green line), and the fine-tuned model (orange line) for Li–Li, Li–Si, and Si–Si pairs, respectively.

Building on this structural fidelity, we further evaluated kinetic accuracy by comparing lithium migration barriers computed with the nudged elastic band (NEB) method.^54^ Diffusion pathways identified from AIMD trajectories were relaxed at the DFT level and then recomputed using both the foundation and fine-tuned MLFFs. Figure 3 shows representative migration paths and their corresponding energy profiles for Li_12_Si_7_ and Li_13_Si_4_. The foundation model systematically underestimates and occasionally overestimates the migration barriers, reflecting its emphasis on broad transferability at the expense of localized precision. In contrast, the fine-tuned MLFFs reproduce DFT-calculated barriers within 2–5% error, in some cases yielding nearly indistinguishable profiles (see the SI). In Li_13_Si_4_, a dominant one-dimensional diffusion channel was identified along a crystallographic axis, with its barrier precisely captured by the fine-tuned model (Figure 5). Accurate barriers are essential because diffusivities follow Arrhenius-type scaling, where even modest deviations propagate exponentially. By matching DFT-calculated barriers across multiple migration paths in both Li_12_Si_7_ and Li_13_Si_4_, the fine-tuned MLFFs establish a reliable foundation for transport modeling. This accuracy ensures that lithium jump statistics extracted from long MLFF simulations can be coarse-grained into Markov state models (MSMs) without systematic bias in the underlying energetics. With barrier-level agreement verified, we proceed to use these MLFFs to generate size-converged, nanosecond-scale trajectories for lithium transport analysis.

**Figure 3:**
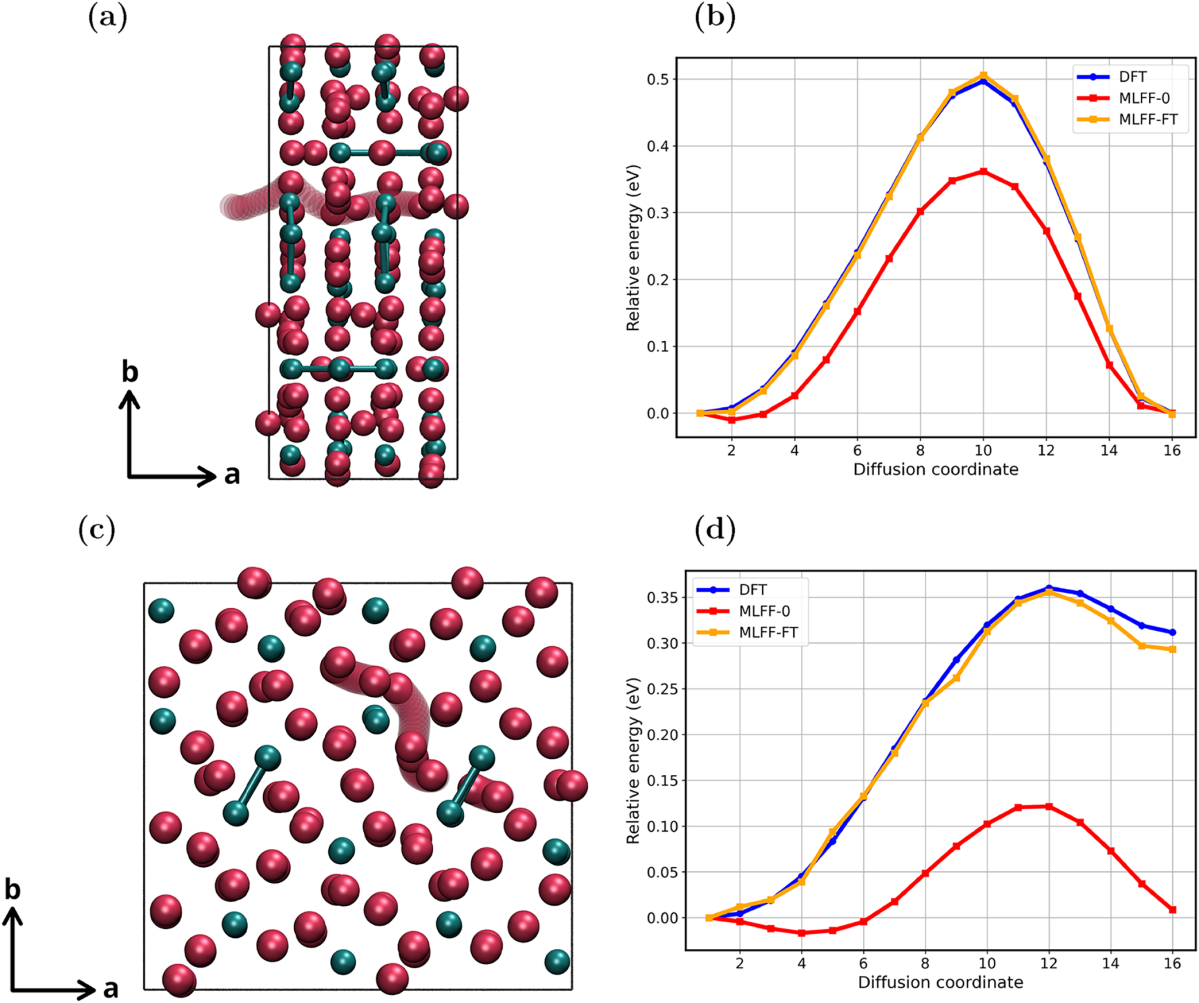
Representative lithium migration pathways and activation energies. Panels (a, c) show selected migration paths in Li_12_Si_7_ and Li_13_Si_4_, respectively. Panels (b, d) show corresponding NEB energy profiles computed using DFT (blue), the MACE foundation model (red), and the fine-tuned model (orange).

### 3.1 Lithium Diffusion and Finite-Size Effects

Having established that the fine-tuned MLFFs reproduce both static energetics and migration barriers with near-DFT accuracy, we next employ them to explore lithium diffusion dynamics over extended time scales and to quantify finite-size effects on the computed transport coefficients. The mean square displacements (MSDs) for Li_12_Si_7_ and Li_13_Si_4_ obtained from MLFF and AIMD simulations are shown in Figure 4a. MSDs from both methods are in very good agreement for both compounds, further confirming the accuracy of the MLFFs.

**Figure 4:**
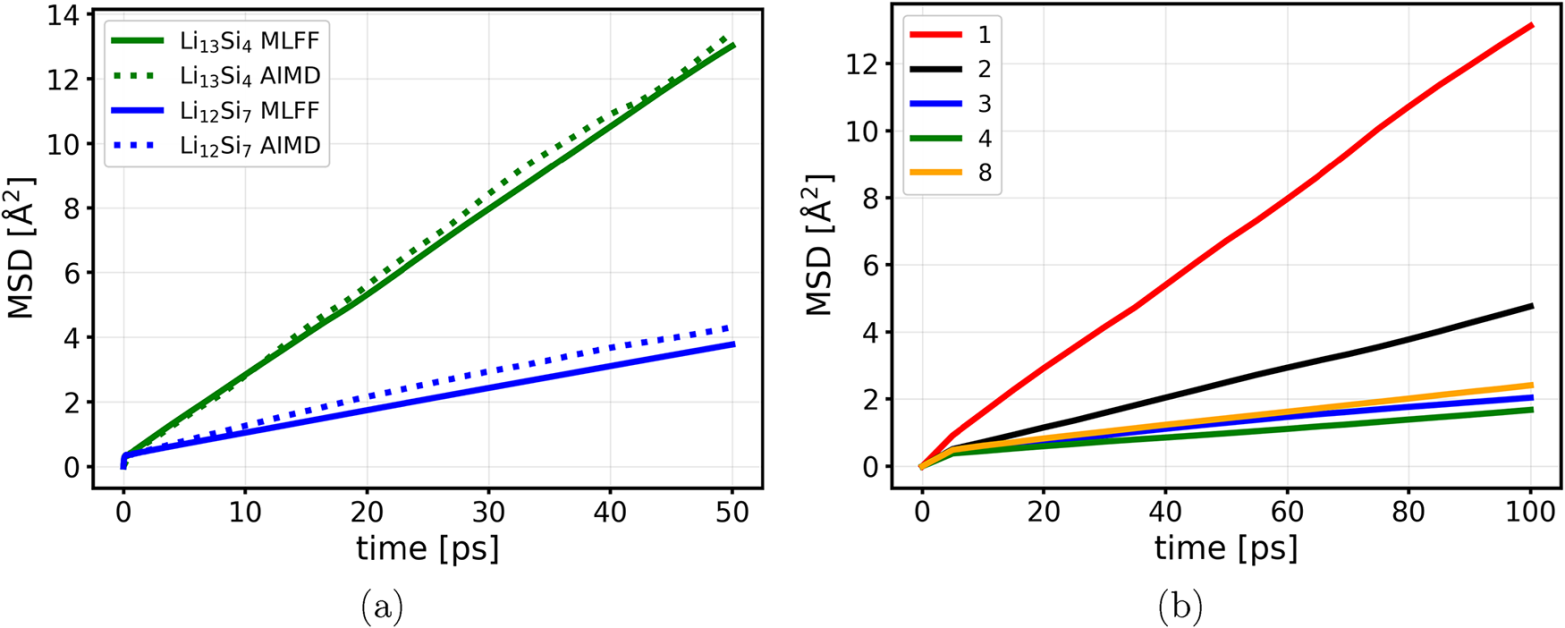
(a) MSD from AIMD and MLFF simulations (b) Convergence of the diffusion coefficient (MSD slope) for Li_13_Si_4_ with respect to system size in *x*-direction from MLFF simulations. Values 1, 2, 3, 4, and 8 in the legend correspond to 1-, 2-, 3-, 4-, 8-fold increased supercell dimension in *x*-direction. Apparent diffusivities computed in AIMD–accessible supercells exhibit a pronounced finite–size bias, whereas larger MLFF supercells approach the asymptotic, size–independent limit.

Li_12_Si_7_ and Li_13_Si_4_ were selected because, compared to other crystalline lithium silicides such as Li_15_Si_4_, LiSi, and Li_17_Si_4_, they exhibit exceptionally low migration barriers for lithium diffusion even in defect-free systems. As a result, converged diffusion coefficients can be obtained on AIMD time scales, allowing the MSDs of these compounds to serve as meaningful benchmarks for evaluating the accuracy of the MLFFs.

However, the situation becomes more complex when examining the directional components of the MSD. In Li_13_Si_4_, lithium mobility is dominated by diffusion along one-dimensional channels oriented along the *x* direction, whereas diffusion along the *y* and *z* directions constitutes a rare event on AIMD time scales. Figure 10 compares the MSD along the *z* direction obtained from short AIMD and long MLFF simulations. Meaningful slopes of the MSD and therefore reliable diffusion coefficients are accessible only from the MLFF simulations. The diffusion coefficient along the *x* direction $\left(0.02\;\frac{{Å}^{2}}{\mathrm{ps}}\right)$ is approximately 400 times larger than that along the *z* direction $\left(5\cdot 10^{-5}\;\frac{{Å}^{2}}{\mathrm{ps}}\right)$ . Lithium diffusion in the *y* and *z* directions can also be detected in AIMD simulations, as reported in ref 41 but only at significantly elevated temperatures that help to overcome the higher migration barriers.

Motivated by the quasi-one-dimensional diffusion channel in Li_13_Si_4_ (cf. Figure 5),^41^ we performed a systematic size study that is impractical with AIMD but feasible with MLFFs. Elongating the supercell along the transport axis reveals that small boxes (e.g., 1 × 1 × 1) overestimate the MSD slope due to repeated self-encounters across periodic images. As the box is extended (e.g., 2 × 1 × 1, 6 × 1 × 1, 8 × 1 × 1), the apparent diffusion coefficient *D* decreases and the MSD curves converge to an asymptotic limit, indicating size convergence (Figure 4b). Hence, (i) AIMD-level diffusivities in anisotropic networks are biased high by finite-size effects, and (ii) MLFFs enable the larger simulation cells required to obtain size-independent transport coefficients. These converged trajectories provide the well-sampled jump statistics used to construct the Markov models discussed below.

**Figure 5:**
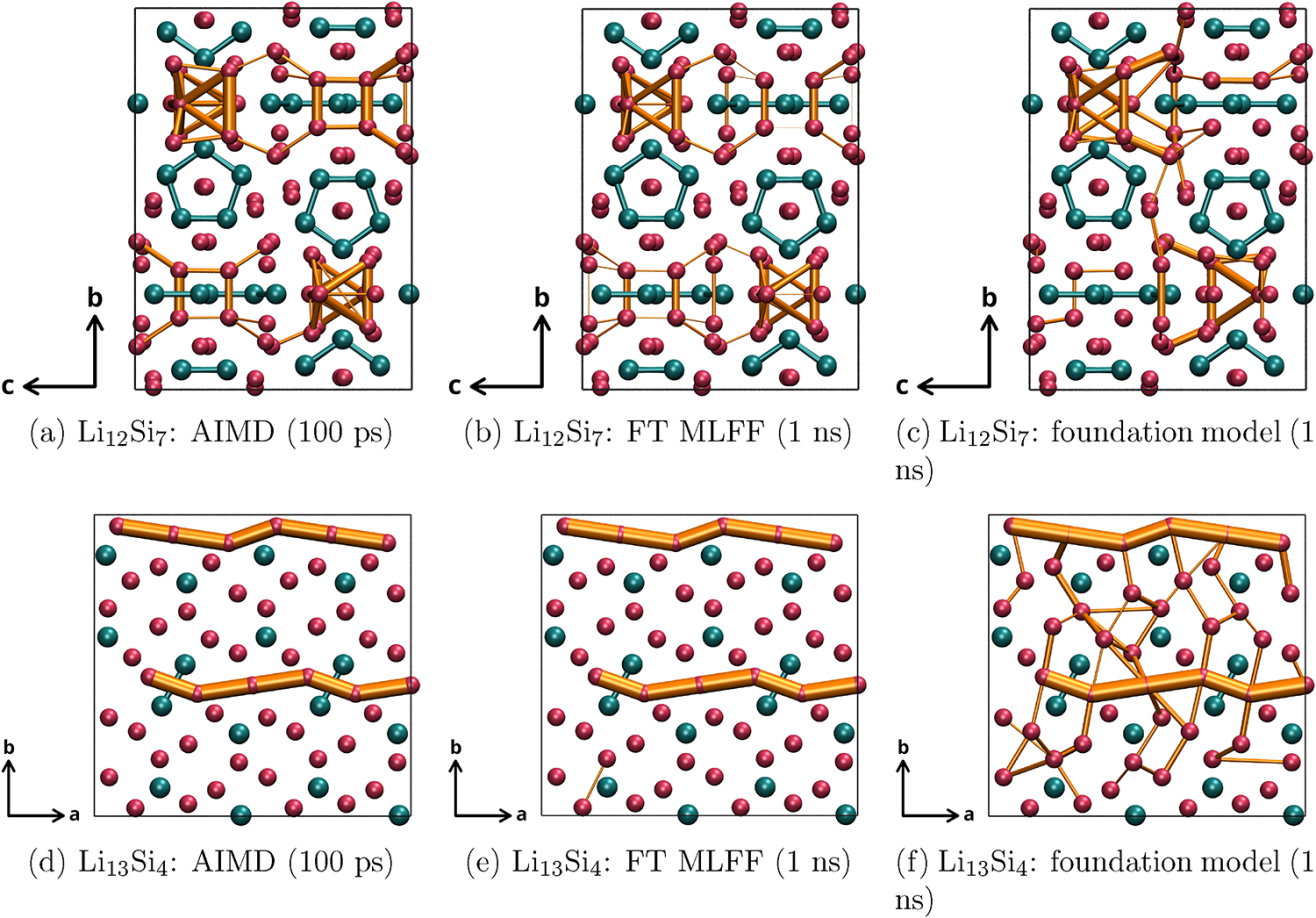
Average Li^+^ jump frequency maps at 500 K. Top row: Li_12_Si_7_ with (a) AIMD (100 ps), (b) fine-tuned MLFF (1 ns), (c) foundation model (1 ns). Bottom row: Li_13_Si_4_ with (d) AIMD (100 ps), (e) fine-tuned MLFF (1 ns), (f) foundation model (1 ns). AIMD time scales are too short to converge jump statistics, while the foundation model overestimates rates; fine-tuned MLFFs recover AIMD-observed channels and sample rare events.

#### 3.1.1 Lithium Jump Statistics and Markov State Models

Transition matrices $M^{\tau }$ were sampled from the MLFF trajectories according to eq 5. By using the crystallographic Li positions as lattice sites, continuous MLFF trajectories are mapped onto discrete networks. A detailed protocol for the construction of the transition matrices was given in Section 2.2.4.

Figure 5 shows representative jump networks, i.e., visualizations of the transition matrices $M^{50\mathrm{fs}}$ for Li_12_Si_7_ and Li_13_Si_4_, extracted from AIMD (100 ps), fine-tuned MLFF (1 ns), and foundation-model (1 ns) trajectories. Line thickness encodes hop frequency. AIMD and fine-tuned MLFFs are in good agreement, which is expected as energy barriers for lithium jumps and diffusion coefficients were already in good agreement. The dominant 1D-diffusion in Li_13_Si_4_ becomes immediately apparent from Figure 5d,5e. The jump networks resulting from the foundation model are shown in Figure 5c,5f, and they are artificially dense due to underestimated migration barriers.

In addition, we systematically analyzed the transition matrix $M^{\tau }$ for different lag times τ in terms of its eigenvalues and eigenvectors. The absolute value of each eigenvalue lies between 0 and 1. The eigenvalue spectrum of $M^{\tau }$ for Li_13_Si_4_ at τ ∈ {0.06, 5, 80 ps} is shown in Figure 6a. Since the simulation cell contained 156 Li sites, 156 eigenvalues were obtained. The eigenvalues can be grouped into two distinct regions: the first 126 are very close to unity, while the remaining 30 are significantly smaller. The largest eigenvalue equals one, and its corresponding eigenvector describes the uniform distribution of lithium atoms across the available lattice sites. This state represents the equilibrium distribution expected from the MD trajectory. Eigenvectors associated with smaller eigenvalues describe relaxation processes toward this equilibrium distribution.

**Figure 6:**
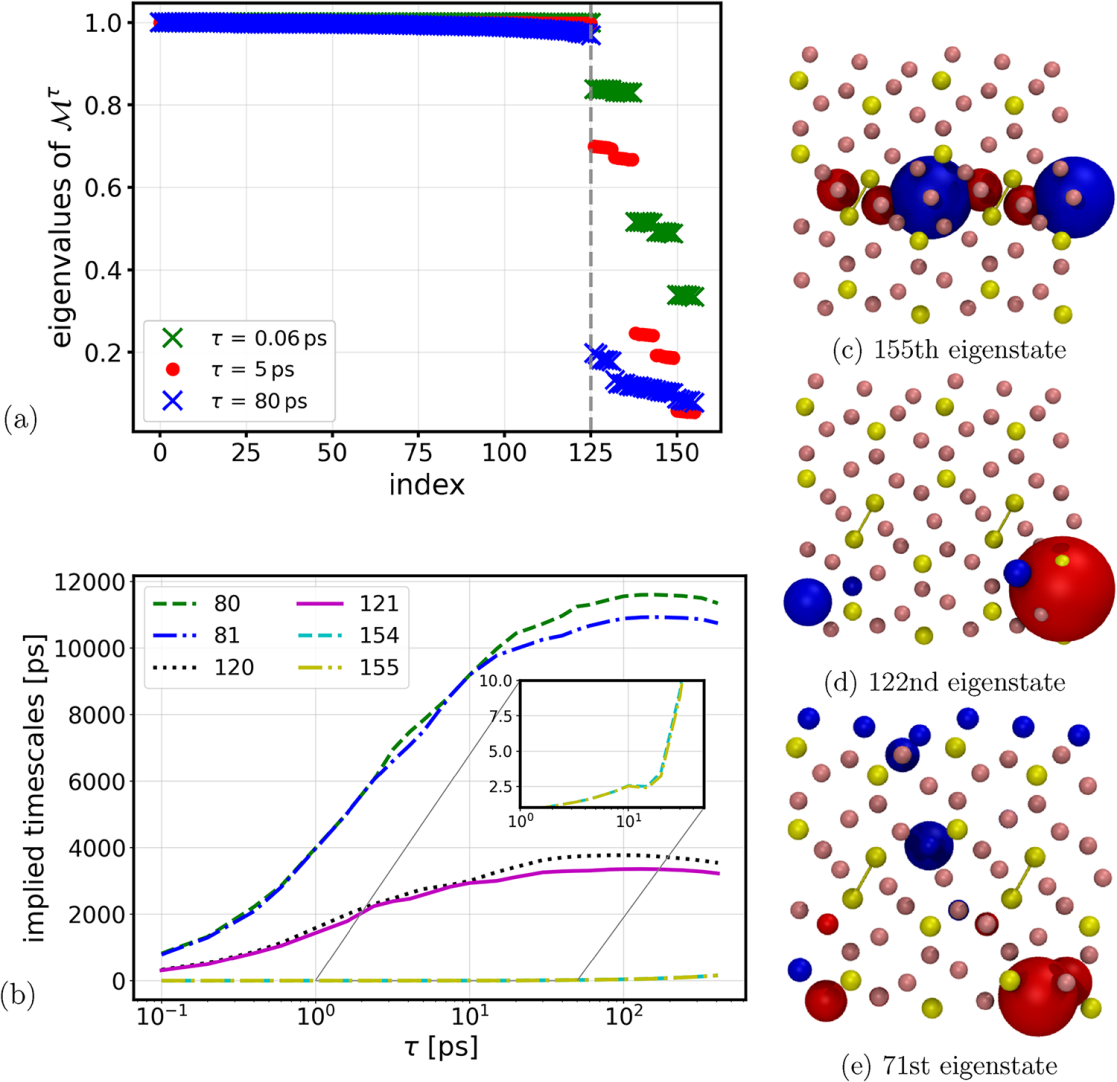
(a) Eigenvalue spectrum of the transition matrix $M^{\tau }$ for Li_13_Si_4_ at τ ∈ {0.06, 5, 80 ps}. (b) Implied time scales $t_{k}\left(\tau \right)=-\frac{\tau }{\ln\lambda_{k}\left(\tau \right)}$ calculated from the eigenvalues of $M^{\tau }$ for different lag times τ. (c–e) Visualization of selected eigenstates.

Three representative eigenvectors of the transition matrix $M^{80\mathrm{ps}}$ for Li_13_Si_4_ are visualized in Figure 6c–e. For our specific choice of crystallographic Li positions as lattice sites, these eigenvectors can be correlated with lithium diffusion pathways. Eigenvectors corresponding to eigenvalues significantly smaller than one (index ≥ 126) exhibit nonzero components only for Li atoms located within a single one-dimensional diffusion channel. A particular example is the 155th eigenstate shown in Figure 6c. These delocalized eigenstates represent fast, “rattling” motions of Li atoms within the one-dimensional channels. In contrast, eigenvectors with indices smaller than 126 are more complex and spatially not only localized in the 1D channels and contribute also to displacements of Li atoms along the *y*- and *z*-directions. Representative examples are the 71st and 122nd eigenstates (Figure 6d,e). The eigenvalues with indices smaller than approximately 126 are significantly greater than the smallest 30 eigenvalues and therefore correspond to relaxation processes on much longer time scales.

In the upper part of Figure 6e (eigenstate 71), an entire one-dimensional diffusion channel appears in a single color (blue). The corresponding eigenvector thus describes processes in which Li atoms initially located outside the channel move into the channel and rapidly acquire nonzero occupation probabilities for all lattice sites within it. Eigenstates with indices between roughly 100 and 126 exhibit a broad distribution of nonzero vector elements in the *x*–*y* plane but remain localized along the *z* direction. For smaller indices (below ∼ 100), the distribution becomes increasingly uniform. Particular examples for these two regimes are eigenstate 71 and 122 in Figure 6.

Because significant spreading along the *z* direction occurs only for eigenstates with small indices, diffusion along *z* must be considerably slower than diffusion in the *x*–*y* plane.

Diffusion along the *y*- and *z*-directions is not an artificial artifact. Li jumps in these directions occur with much lower frequency compared to those along the *x*-direction. Consequently, the corresponding line thicknesses representing Li jump frequencies between lattice sites in Figure 5 are too small to be visible.

Figure 6b shows the implied time scales, defined in eq 3, as a function of lag time τ for six representative eigenvalues. Different eigenvalues exhibit distinct minimal lag times τ_min_, marking the onset of the plateau region in the implied-time scale curves. Smaller eigenvalues correspond to faster relaxation processes and therefore reach their plateau at shorter lag times. In Figure S4–S11, we show the implied time scales for all eigenvalues. The implied time scales associated with the 30 lowest eigenvalues do not exhibit an extended plateau region. However, for the lag times τ relevant for the construction of the MSM, the corresponding implied time scales are already smaller than τ. This indicates that the associated dynamical modes decay within the lag-time interval and are therefore effectively averaged out at the level of the coarse-grained dynamics. Consequently, these fast modes do not prevent the model from becoming approximately Markovian. In other words, even though not all modes display clear plateau formation, the required separation of time scales is still achieved, which allows the MSM to provide a consistent kinetic description of the remaining slow processes.

Finally, the Chapman–Kolmogorov (CK) test [eq 2] was used to determine the minimal lag time τ required for constructing a consistent Markov model. The relative error err­(*n*), defined in eq 6, quantifies the deviation between the directly sampled multilag transition matrix $M_{\mathrm{sampled}}^{n\tau }$ and the Markovian prediction ${\left(M^{\tau }\right)}^{n}$ (Figure 8). We systematically analyzed err­(*n*) as a function of (i) the lag time τ and (ii) the total trajectory length *T*. Lag times of τ = 5 ps (comparable to τ_min_) and τ = 0.05 ps (well below τ_min_) were examined, together with trajectory lengths of 100 ps (typical of AIMD) and 10–30 ns (typical of MLFF-MD).

Note that error plots for different τ share the same *x*-axis (the Markov chain length *n*) but correspond to different physical times *n*τ. For example, a chain length of *n* = 100 represents a physical time of 5 ps for τ = 0.05 and 500 ps for τ = 5 ps.

For long MLFF trajectories (*T* ≈ 10 ns) and lag times near τ_min_, the CK error remains very small. In these cases, transition matrices sampled at τ = 5 ps accurately reproduce Li^+^ dynamics over time intervals up to 128 × τ (∼600 ps) with an error of only ∼15%. In contrast, MSMs constructed from short (100 ps) trajectories exhibit substantially larger errors, highlighting the need for extended sampling to achieve Markovian consistency.

For lag times shorter than τ_min_, we observe comparable errors for transition matrices sampled from both short (100 ps) and long (10–30 ns) trajectories. This indicates that at very short lag times, AIMD-level sampling is already sufficient to capture local transitions. However, the errors for such short lag times (independent from the overall length of the trajectory *T*) display a pronounced nonmonotonic dependence on the chain length *n*, reflecting the breakdown of the Markov assumption and the presence of unresolved memory effects in the underlying dynamics. Consequently, the observed non-Markovian behavior does not originate from insufficient sampling but rather from violating the minimum lag time required for proper state decorrelation, which is imposed by the discretization of the phase space. Figure 7 illustrates how the discretization of the state space imposes a lower bound on the lag time τ required for constructing consistent MSMs.

**Figure 7:**
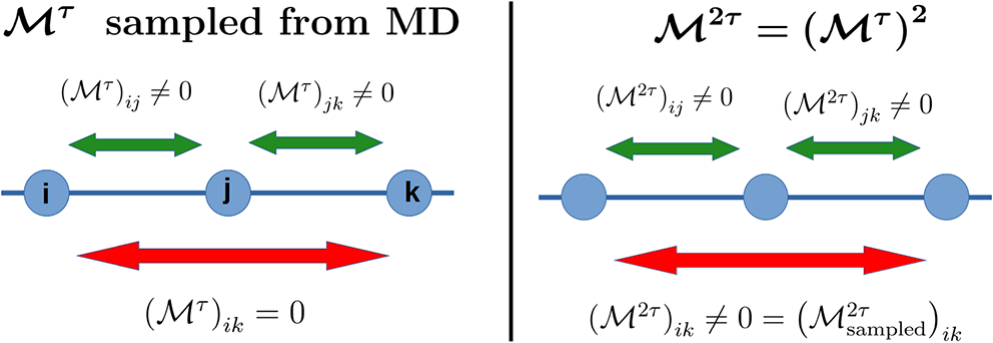
Illustration of how the discretization of the state space imposes a lower bound on the lag time τ required for constructing consistent MSMs. Sampling transition matrices $M^{\tau }$ at very short lag times results in nonzero matrix elements describing Li transfer probabilities between neighboring lattice sites, while matrix elements corresponding to next-nearest neighbors remain zero. The transition matrix $M^{2\tau }$ for the interval 2τ is obtained by squaring $M^{\tau }$ . By construction, this matrix now includes nonzero elements that represent ion transfer probabilities between next-nearest neighbors, even if no such jumps were directly detected in the MD trajectory within the interval 2τ.

In summary, Figures 6 and 8 show that for lag times larger than approximately 5 ps meaningful Markov models can be constructed for crystalline Li_*x*_Si_*y*_ phases using only the small state space defined by the crystallographic lattice sites. On these time scales a separation between fast and slow dynamical processes emerges. The fast modes decay within the lag-time interval and are therefore averaged out in the coarse-grained dynamics, which is a key requirement for the validity of a Markov state model. Consequently, Li motion can be described as effective independent hopping between lattice sites in an effective bath formed by the surrounding Li and Si ions. This behavior is consistent with the diffusion mechanism illustrated in Figure 1a (and Figure 1c for the one-dimensional channel segments of the system).

**Figure 8:**
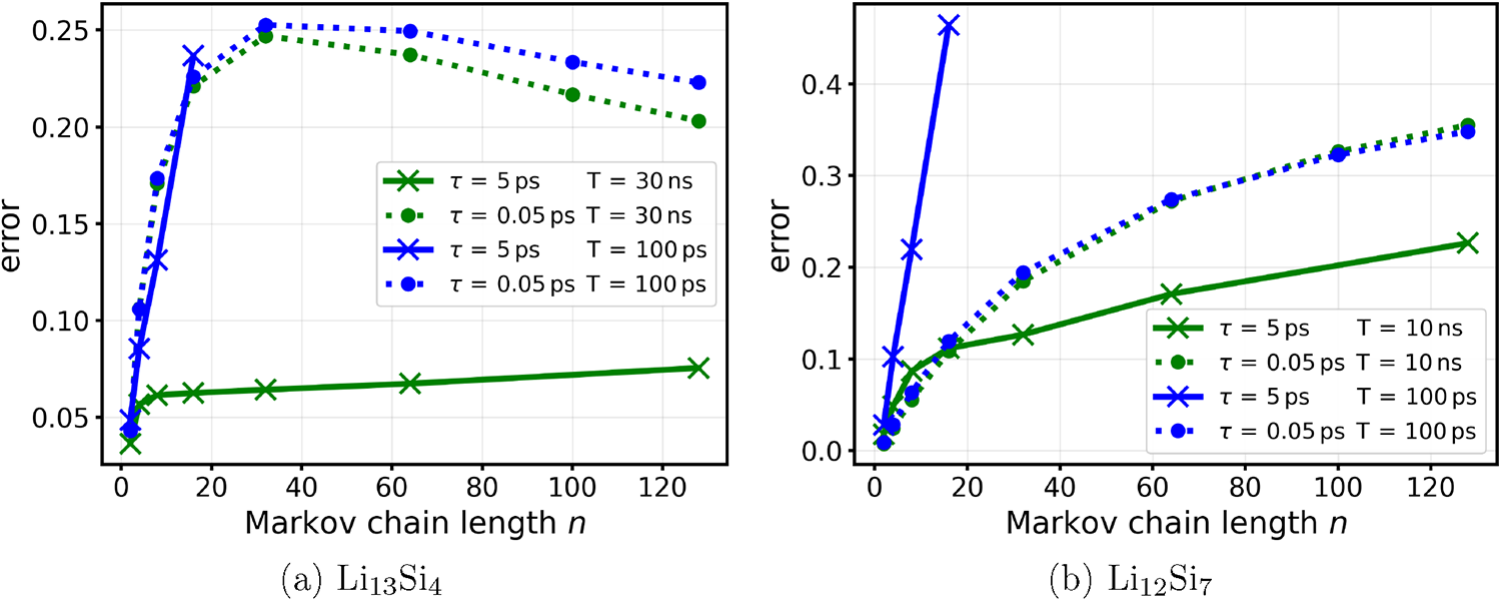
Evaluation of the Markov property using the Chapman–Kolmogorov test for (a) Li_12_Si_7_ and (b) Li_13_Si_4_. The plot compares the directly sampled multilag transition matrix $M_{\mathrm{sampled}}^{n\tau }$ with the Markovian prediction ${\left(M^{\tau }\right)}^{n}$ . The relative error err­(*n*), defined in eq 6, is shown on the *y*-axis as a function of the Markov chain length *n*. The solid blue line is shown up to *n* = 16 because transition matrices can only be sampled for a maximum lag time of τ ·*n* = 5 ps·16 = 80 ps from 100 ps trajectories. We note that the transition matrix constructed at a lag time of 5 ps remains far from the matrix whose rows correspond to the stationary distribution (see Figure S12). This indicates that the system has not yet relaxed to equilibrium and the Markov models constructed at this lag time should therefore be considered meaningful.

For lag times larger than approximately 5 ps, correlations between the motion of different Li ions (as represented by the mechanism in Figure 1b) no longer influence the coarse-grained dynamics.

Additional insight into the short-time correlated motion is provided by the implied-time scale analysis in Figure 6. The fastest processes, associated with the lowest 30 eigenvalues, must decay before the model becomes Markovian because their implied time scales do not exhibit plateau formation. Visualization of these modes for Li_13_Si_4_ reveals a “rattling” motion of Li atoms within the one-dimensional channels, which reflects short-range correlations caused by the exclusion of multiple Li ions from the same lattice site (cf. Figure 1b).

We therefore conclude that Li–Li correlations are responsible for the non-Markovian behavior observed at lag times below 5 ps, and that the eigenvectors associated with the lowest 30 eigenvalues represent signatures of these short-time scale correlated Li motions. This analysis therefore provides a mechanistic interpretation of the MSM spectrum in terms of short-time scale correlated motion and long-time scale effective independent hopping.

In the final step, transport properties were reconstructed by propagating displacements through the MSM according to eq 7. The mean-square displacement of the Li ions was calculated from the Markov chain using eq 7. Figure 9 compares MSDs for Li_12_Si_7_ and Li_13_Si_4_ obtained from MSMs sampled at different lag times and trajectory lengths. MSDs for both compounds were calculated from transition matrices constructed for lag times of τ ∈ {0.5, 5, 20, 80 ps}. Sampling intervals of length τ were obtained from 100 ps trajectories (typical of AIMD) and from 10–30 ns trajectories (typical of MLFF-MD). For τ = 80 ps, transition matrices could only be constructed from the MLFF-MD trajectories.

**Figure 9:**
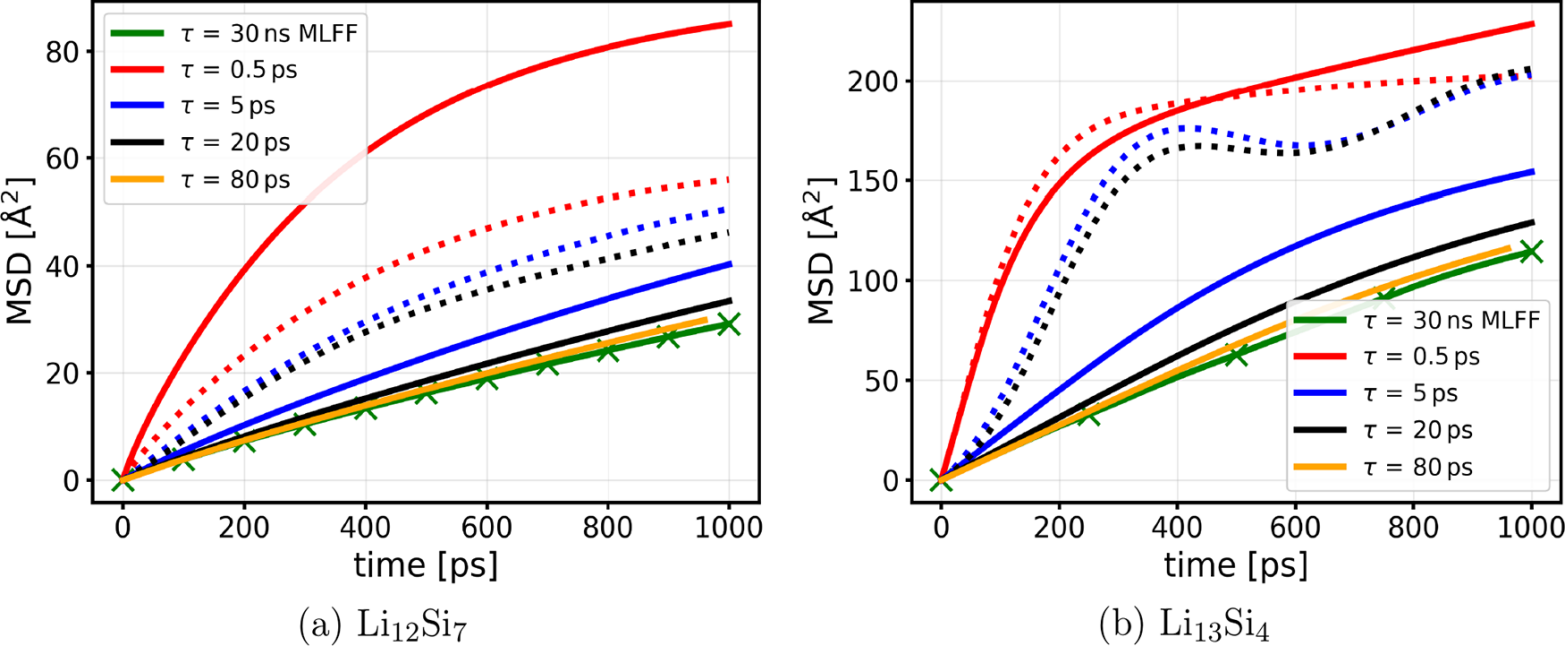
MSD at 500 K for (a) Li_12_Si_7_ and (b) Li_13_Si_4_ from MLFF-MD (green) and MSM reconstructions from transition matrices $M^{n\tau }$ sampled with lag time τ. Solid (dashed) lines are obtained from transition matrices sampled from MLFF simulation of total length 10 ns (100 ps). MSMs trained on long trajectories for minimal lag times τ > 5 ps reproduce long-time behavior, while MSMs from 100 ps MD and shorter lag times deviate. Calculation of MSD from transition matrices according to eq 7 does not take into account periodic images. Therefore, maximum displacement is half of the box dimensions, leading to a plateau for very long time scales.

All MSMs trained on 100 ps trajectories overestimate long-time transport. Similarly, MSMs constructed with lag times shorter than 5 ps deviate significantly from direct MLFF-MD results, indicating a breakdown of the Markovian approximation. Only MSMs sampled from 10–30 ns MLFF trajectories and lag times greater than 5 ps accurately reproduce the MSDs up to 1 ns. The agreement between MSMs and direct MLFF results improves systematically with increasing lag time and is good, very good, and excellent for τ values of 5, 20, and 80 ps, respectively.

As a representative example demonstrating the advantage of the extended time scales accessible through MLFF and MSM simulations compared to AIMD, Figure 10 shows the MSD along the *z*-direction for Li_13_Si_4_ obtained from 100 ps AIMD, 30 ns MLFF, and MSM simulations. The MSM was constructed from the transition matrix $M^{80\mathrm{ps}}$ sampled from the long MLFF trajectory. For very short time scales (<10 ps), the MSD offset agrees well between the AIMD and MLFF simulations. However, the AIMD-derived MSD remains constant at longer times, whereas the slopes of the MSD curves from MLFF and MSM simulations show excellent agreement in this regime. The inability of AIMD to capture the correct long-time slope—and the strong consistency between the MLFF and MSM results—becomes even more evident when fluctuations around the crystallographic lattice sites are removed by projecting the Li positions at each time step onto the nearest lattice site. The “projected” MSD obtained from AIMD is exactly zero at all times, indicating the absence of Li jumps between lattice sites with different *z*-coordinates, whereas the projected MSDs from the MLFF and MSM simulations are nearly indistinguishable.

**Figure 10:**
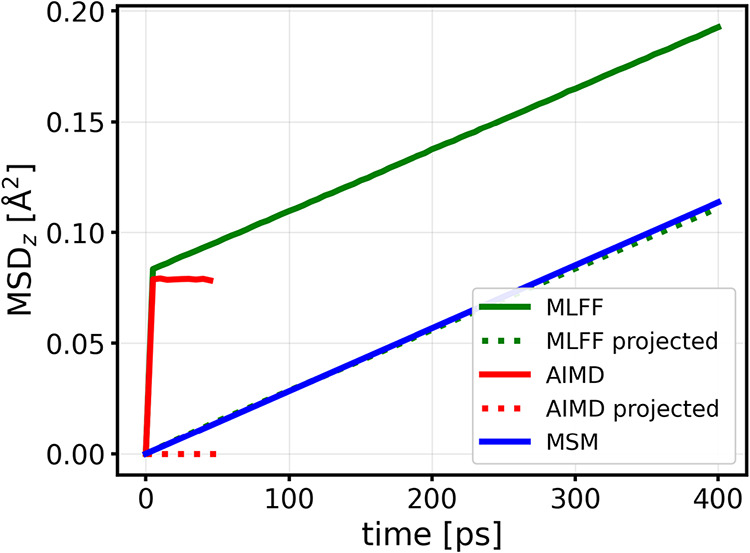
*Z*-component of the MSD of Li_13_Si_4_ obtained from 100 ps AIMD, 30 ns MLFF-MD, and MSM simulations. For the “projected” MSDs, the positions of the Li atoms were mapped to the nearest lithium lattice sites. This procedure removes the contributions from local fluctuations around crystallographic lattice sites and is implicitly included in the Markov models by construction. Meaningful diffusion coefficients can only be obtained from the MLFF and MSM simulations.

In summary, reliable MSMs for Li–Si systems require lag times greater than 5 ps and training trajectories on the order of tens of nanoseconds. Once constructed, these models accurately predict the collective dynamical evolution of all Li^+^ ions through simple matrix–vector propagation. This approach extends accessible temporal and spatial scales and enables simulation of lithium mobility on the microsecond time scale. Such scales are sufficient to augment existing continuum and multiscale models of silicon anodes with a physically grounded description of lithium diffusion within these materials.^55–59^

Previous multiscale approaches have primarily focused on chemomechanical modeling of lithiation-induced failure in high-volume-change anodes, often employing diffusion coefficients that do not explicitly depend on the local lithium concentration. As a next step, we aim to couple MSMs sampled at different lithium concentrations—particularly from amorphous Li–Si systems—to derive concentration-dependent diffusion models applicable at the electrode scale.

### 3.2 Outlook: Extension of the Workflow to Amorphous Phases

In principle, the present fine-tuning approach for the MACE foundation model can also be applied to amorphous Li_*x*_Si. However, compared to crystalline phases, amorphous systems exhibit a much larger variety of local environments even at fixed coordination, which implies that the training set must be correspondingly more diverse. This has been demonstrated in several studies on ML interatomic potentials for amorphous materials. For amorphous Li–Si, Artrith et al. and Onat et al. constructed neural-network potentials for Li_*x*_Si alloys and showed that accurate description of amorphous phases requires extensive sampling of structurally distinct configurations, but that energies and forces for both crystalline and amorphous states can be reproduced with similar accuracy once the training set is sufficiently rich.^60,61^ Xu et al. reached analogous conclusions for a deep-learning potential that simultaneously describes crystalline and amorphous Li–Si.^33^ For other amorphous materials (a-Si, a-Si/H, amorphous carbon, silicate and ZIF glasses), machine-learned potentials such as GAP, DeePMD, and more recently MACE-type models have likewise been shown to achieve near-DFT accuracy for energies, forces, and structural observables, provided that the training data includes melt–quench trajectories and broad coverage of local environments.^62–66^ These works suggest that extending our fine-tuned MACE model to amorphous Li_*x*_Si should be feasible, but will likely require substantially more (and more heterogeneous) ab initio training data than in the purely crystalline case. In a proof-of-principle study, we have already tested this hypothesis for two amorphous phases, Li_300_Si_100_ and Li_134_Si_268_. We generated training data from two 10 ps AIMD trajectories of these amorphous systems at 500 K and extracted 2000 equidistant snapshots for fine-tuning. Across both compositions, the fine-tuned models achieved energy errors below 5 meV atom^–1^ and force errors below 55 meV Å^–1^ on test sets obtained from a train–validation–test split of the available data. Details are shown in the SI. The resulting RDFs from MD simulations using these force fields are shown in Figure 11 and exhibit very good agreement with AIMD reference data. Importantly, we also benchmarked the quality of the force fields for describing lithium migration energy profiles. Following characteristic lithium displacements identified in the AIMD trajectories, we extracted the corresponding initial and final configurations and performed NEB calculations. The resulting DFT energy profiles, compared with the MLFF-predicted energies, are shown in Figure 11 and the SI. The agreement between DFT and MLFF is very good, and fine-tuning substantially improves the correspondence of the profiles. Compared to the excellent agreement achieved for the crystalline phases, the performance for the amorphous phases is slightly reduced, and the energy and force errors are moderately higher. This behavior is expected, as MLFF training for amorphous materials is inherently more challenging due to the significantly larger configurational diversity. Furthermore, in this proof-of-concept study, the AIMD trajectories used for the amorphous phases were an order of magnitude shorter than those used for the crystalline systems. Despite these limitations, the efficiency of the fine-tuning procedure is remarkable, especially considering that no active-learning strategy was employed to assemble the training data—an approach that has been necessary in previous studies aiming to construct accurate MLFFs for amorphous systems.

**Figure 11:**
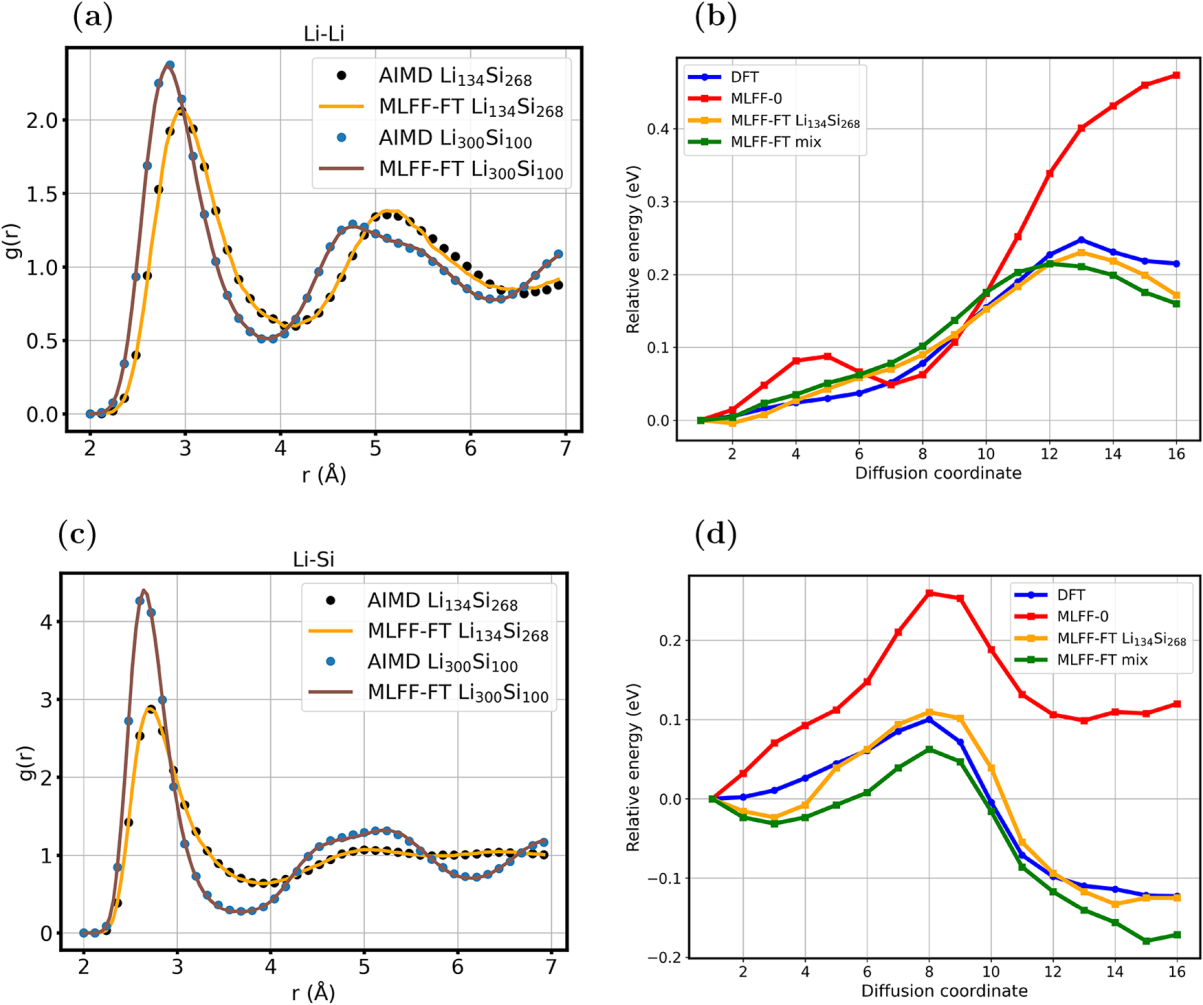
Radial distribution functions (RDFs) and representative lithium migration pathways for two amorphous phases. Panels (a, c) show Li–Li and Li–Si RDFs obtained from AIMD and simulations using fine-tuned MLFFs. The fine-tuned force fields were obtained by refining the foundation model using compound-specific training data. The corresponding Si–Si RDFs are provided in the Supporting Information. Panels (b, d) show NEB energy profiles for Li_134_Si_268_ and Li_300_Si_100_ computed using DFT, the pretrained foundation model, and fine-tuned models. Two fine-tuning strategies are compared: models trained on compound-specific data and models trained on mixed data from different amorphous phases. In all cases, fine-tuning improves the agreement of the MLFF predictions with the DFT reference compared to the foundation model. Additional energy profiles are reported in the Supporting Information.

Based on these proof-of-principle results, we expect that fine-tuning using training data extracted from several 100 ps AIMD trajectories of amorphous Li_*x*_Si with varying Li concentrations will yield highly accurate MLFFs. Importantly, the force fields obtained in the present proof-of-principle study already exhibit sufficient accuracy for use in production simulations.

## 4 Conclusions

We have established a robust multiscale framework that bridges quantum-accurate atomistic simulations and mesoscale lithium transport in battery materials by integrating *ab initio* molecular dynamics, machine-learned force fields, and Markov state models. This combination overcomes the temporal and spatial limitations of conventional atomistic simulations while retaining first-principles fidelity. Fine-tuned equivariant MLFFs reproduce DFT migration barriers within a few percent, enabling nanosecond-scale simulations in large supercells that reveal anisotropic and collective lithium motion inaccessible to AIMD. These extended trajectories provide statistically converged jump networks from which MSMs can be constructed to describe long-time lithium dynamics through simple stochastic propagation. The resulting models quantitatively recover diffusivities and mean-square displacements over nanosecond time scales and markedly reduce statistical uncertainty compared with direct molecular dynamics. Finite-size effects that bias diffusivities in small AIMD boxes are eliminated, yielding size-independent transport coefficients matching close with experimental magnitudes. While demonstrated here for defect-free crystalline Li–Si phases, the AIMD → MLFF → MSM workflow is general and transferable to amorphous or defect-rich electrodes, interfaces, and solid electrolytes. It provides a pathway to incorporate concentration-dependent and anisotropic diffusion into continuum-scale battery models, offering a physically grounded alternative to empirical transport parameters. More broadly, this approach demonstrates how machine learning can extend the reach of first-principles simulations toward mesoscopic regimes, providing a practical route for predictive studies of ion transport in complex energy materials. In future work, we aim to apply this framework across a broader range of lithium silicide compositions, temperatures, and defect configurations to systematically assess how structure, composition, and concentration influence lithium mobility within the Li–Si system.

## Supplementary Material



## Data Availability

The MACE models, simulation scripts, and data sets used in this study will be made publicly available upon publication via an open-access repository (e.g., Materials Cloud or NOMAD).

## Notes

Details of the fine-tuning procedure for foundational machine-learned force fields (MLFFs); validation of MLFF predictions against DFT calculations including radial distribution functions and migration energy profiles; structural and kinetic benchmarks of MLFF simulations; analysis of implied time scales and eigenvalue spectra of the Markov state models; discussion of the dimension of the dynamical state space under different ion-transport mechanisms; verification that the sampled transition matrix differs from the equilibrium matrix; additional methodology and validation results for fine-tuned MLFFs applied to amorphous Li_*x*_Si_*y*_ phases, including computational details, force and energy error analysis, and additional structural and kinetic benchmarks (PDF)
