# Supplementary material for: Bridging Atomistic and Mesoscale Lithium Transport via Machine-Learned Force Fields and Markov State Models
Source: J Chem Theory Comput. 2026 May 20;22(11):5373–87. doi: 10.1021/acs.jctc.5c02035 (PMC13255252; doi:10.1021/acs.jctc.5c02035)
Supplement: Supplementary file 1 [file ct5c02035_si_001.pdf]

Supporting Information: Bridging Atomistic and Mesoscale Lithium Transport via Machine-Learned Force Fields and Markov State Models

Muhammad Nawaz Qaisrani,† Christoph Kirsch,‡ Aaron Flötotto,† Jonas Hänseroth,† Jules Jean Max Oumard,† Daniel Sebastiani,‡ and Christian Dreßler*,†

†*Ilmenau University of Technology, Theoretical Solid State Physics, Weimarer Straße 32, 98693 Ilmenau, Germany*

‡*Martin-Luther-University Halle-Wittenberg, Institute of Chemistry, Theoretical Chemistry, Von-Danckelmann-Platz 4, 06120 Halle (Saale), Germany*

E-mail: christian.dressler@tu-ilmenau.de

1 MLFF Model creation and validation

1.1 Fine-Tuning of foundational MLFFs

Machine-learned force fields were developed within the MACE framework,[S1] implemented via the MACE Python package (v0.3.10). We initialized from the publicly available MACE-MP-0 foundation model and fine-tuned it on system-specific DFT reference data extracted from AIMD trajectories.[S2,S3]

Fine-tuning was performed using stochastic gradient descent with a learning rate of 0.01 for 200 epochs and a batch size of 5. The loss function combined energy and force con-

tributions with a weighting ratio of 0.1:10, respectively. For each system, two models were trained: one on a reduced dataset (200 frames) and one on an extended dataset (2000 frames), allowing evaluation of training-set size effects on predictive accuracy.

The fine-tuning protocol was carried out using the workflow implemented in the aMACEing_toolkit package.[S4]

1.2 MLFF Models performance against DFT simulations

Table S1 compares the root-mean-square errors (RMSE) in total energies and atomic forces. The training datasets were constructed for $Li_{12}Si_7$ and $Li_{13}Si_4$ from *ab initio* molecular dynamics (AIMD) simulations, using 200 and 2000 equally spaced frames extracted from 100 ps trajectories. The fine-tuned MLFFs achieve energy errors below 2 meV atom^{-1} and force errors below 30 meV Å^{-1} for both systems. Increasing the training dataset from 200 to 2000 configurations further reduces both metrics, confirming smooth learning behavior without signs of overfitting.

The test set was constructed by selecting 100 equally spaced frames from the extended MD trajectories generated using the fully fine-tuned MACE models. Forces and energies calculated for these frames via DFT were compared to those predicted by various MACE models. We observed energy errors below 2 meV atom^{-1} and force errors below 50 meV Å^{-1} for both systems. Detailed information on the test set errors are given in Table S2.

Table S1: Root Mean Square Errors (RMSE) in energy (E) and forces (F) for systems $Li_{12}Si_7$ and $Li_{13}Si_4$ for two training data set size with 200 and 2000 frames. Energies are reported in meV/atom and forces in meV/Å.

**System**	**Training data set**	**RMSE E (meV/atom)**	**RMSE F (meV/Å)**
$Li_{12}Si_7$	200	1.5	29
$Li_{12}Si_7$	2000	0.5	22.5
$Li_{13}Si_4$	200	1.9	18.6
$Li_{13}Si_4$	2000	0.6	14.2

Table S2: Table comparing the predicted energies (E) and forces (F) from fine-tuned MACE models (FT) with explicit DFT calculations for 100 frames obtained from the 10 ns ($Li_{12}Si_7$) and 30 ns ($Li_{13}Si_4$) trajectories generated by MACE. The selected snapshots are independent of the training data, ensuring an unbiased evaluation. Each row represents a different version of the MACE model (see main text for details). The comparison includes root mean square error (RMSE) metrics for energies and forces, respectively.

	**RMSE E** **meV^{-1} atom^{-1}**	**RMSE F** **meV^{-1} Å^{-1}**	**relative F RMSE** **%**
Foundation $Li_{13}Si_4$	177203	136	28
FT 200 $Li_{13}Si_4$	1.8	29	6.1
FT 2000 $Li_{13}Si_4$	0.9	22	4.6
Foundation $Li_{12}Si_7$	163825	219	41
FT 200 $Li_{12}Si_7$	1.8	48	9.1
FT 2000 $Li_{12}Si_7$	0.2	35	6.7

1.3 Structural and kinetic benchmarks for MLFFs

The accuracy of the MLFFs was assessed against both structural and kinetic benchmarks. Structural fidelity was quantified by computing radial distribution functions (RDFs) $g(r)$ from MLFF trajectories and comparing them with AIMD reference data. RDFs were evaluated for all relevant atomic pairs (Li–Li, Li–Si, and Si–Si), with emphasis on reproducing both peak positions and intensities. Kinetic validation was performed by calculating lithium migration barriers using the climbing-image nudged elastic band (CI-NEB) method. Diffusion pathways were identified from AIMD trajectories, and corresponding initial and final states were optimized with DFT at the BLYP level. MLFF-predicted barrier heights were then compared directly against DFT results, providing a stringent test of the models' ability to reproduce the energetics governing lithium transport.

1.3.1 Radial distribution functions for $Li_{13}Si_4$

Figure S1 compares the Radial distribution functions (RDFs) obtained from MLFF molecular dynamics trajectories of $Li_{13}Si_4$ system to AIMD reference data at 500 K, complementing the $Li_{12}Si_7$ results in the main text. The fine-tuned MLFF reproduces all major pair correlations, including the Si–Si and Li–Si peak positions and amplitudes. The long-range decay of $g(r)$

is consistent with AIMD, indicating that the model captures both local coordination and extended order.

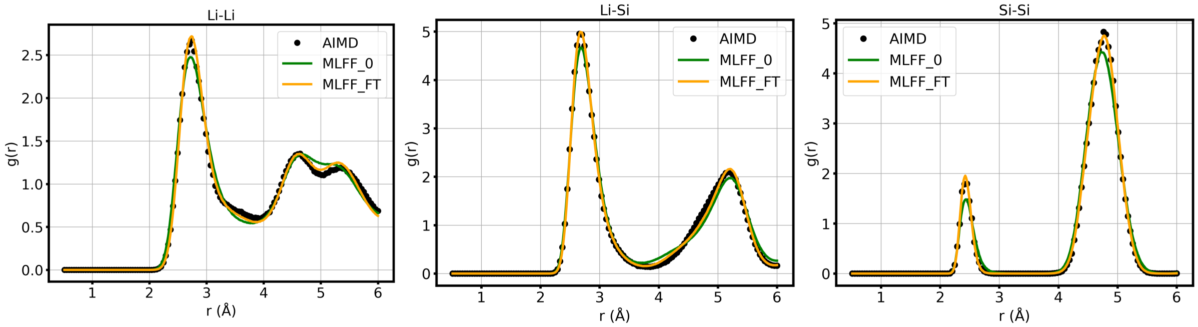

Figure S1: Radial distribution function g(r) obtained from aimd (in black filled spheres) and different MACE models of $Li_{13}Si_4$ computed at 500K.

1.3.2 Nudged elastic band calculations

Figure S2 and Figure S3 illustrates representative several lithium migration pathways identified from AIMD trajectories in both $Li_{12}Si_7$ and $Li_{13}Si_4$, computed using the nudged elastic band (NEB) method. Across all tested paths, the fine-tuned MLFF reproduces DFT activation energies within 2–5 %, whereas the pretrained MACE foundation model shows deviations of up to 15 %. The close agreement of barrier heights ensures that lithium jump statistics derived from long MLFF simulations accurately reflect the underlying DFT energy landscape and can be reliably used for transport modeling.

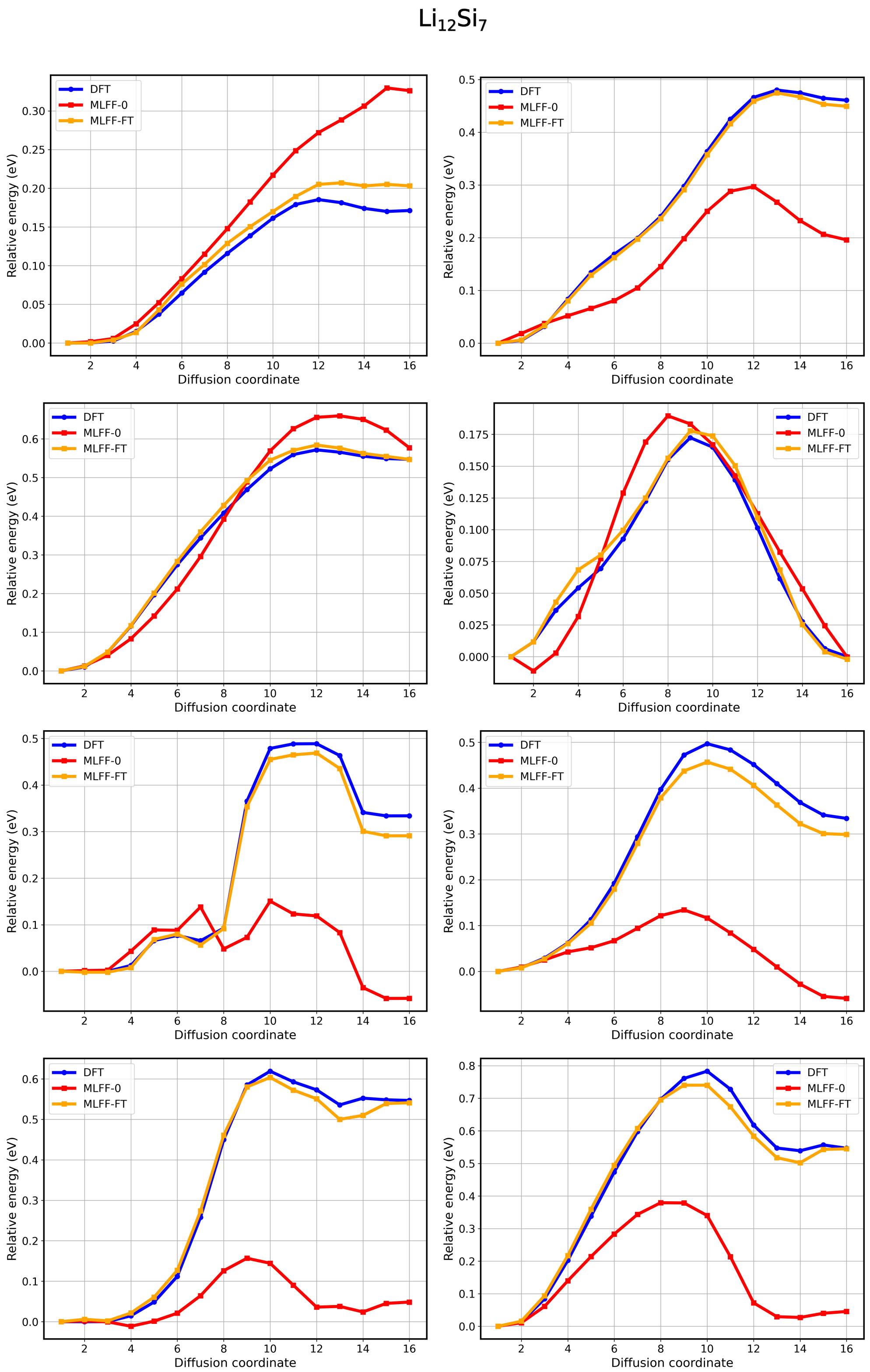

Figure S2: Comparison of several NEB paths of $Li_{12}Si_7$ system, showing that the fine-tuned MLFF accurately reproduces the migration barrier obtained from reference AIMD data, whereas the foundation model either underestimates or overestimates the paths energetics.

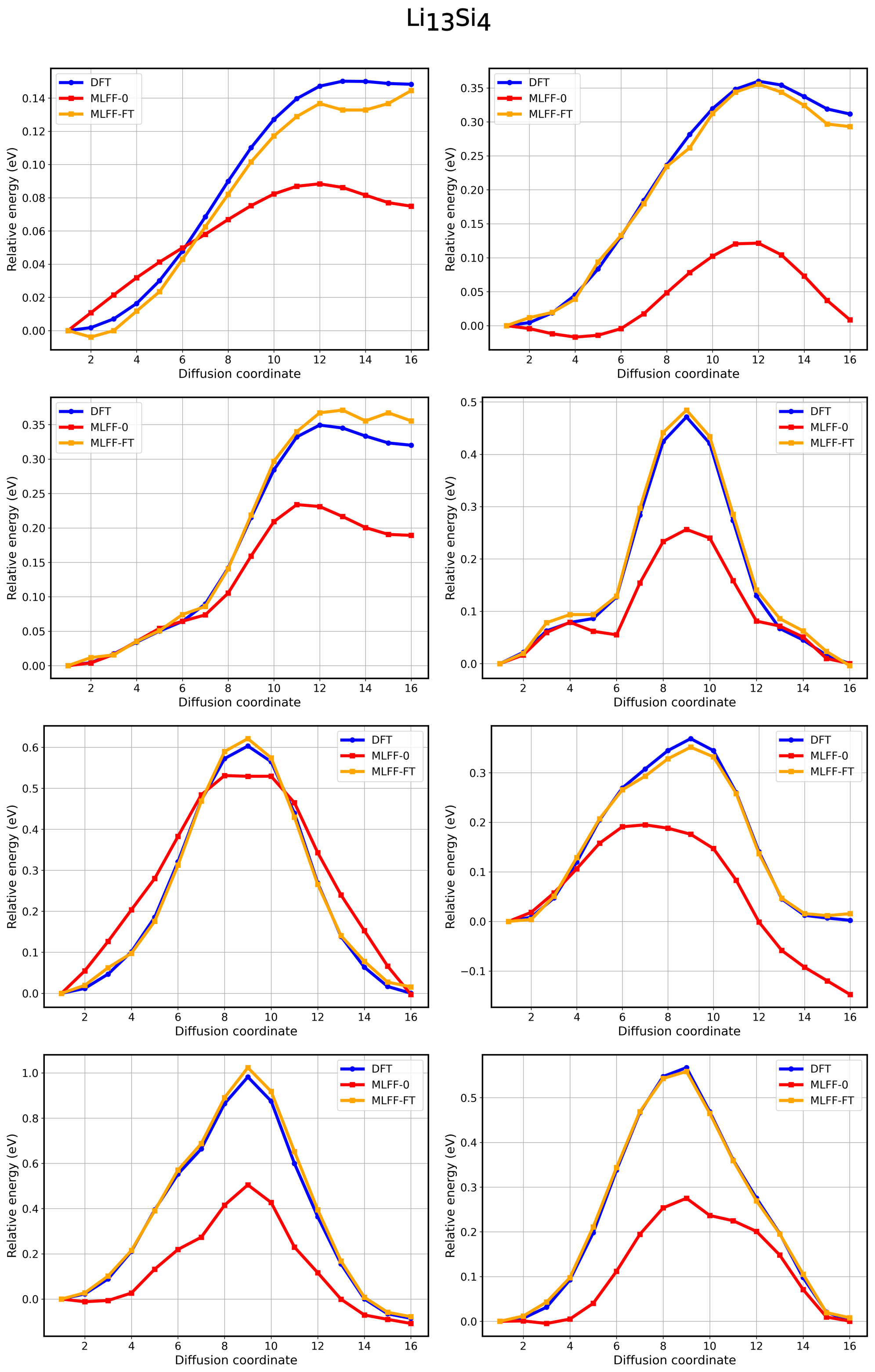

Figure S3: Comparison of several NEB paths of $Li_{13}Si_4$ system, showing that the fine-tuned MLFF accurately reproduces the migration barrier obtained from reference AIMD data, whereas the foundation model either underestimates or overestimates the paths energetics.

1.4 Implied Timescales for Eigenvalues

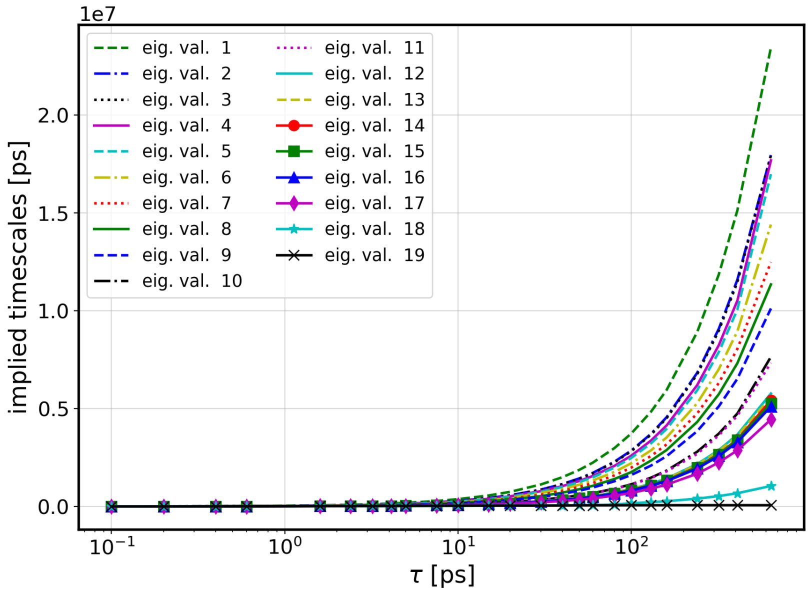

Figure S4: Implied timescales $t_k(\tau) = -\frac{\tau}{\ln \lambda_k(\tau)}$ for $Li_{13}Si_4$ calculated from eigenvalues of $\mathcal{M}^\tau$ for different lag times τ. The implied timescale for the first eigenvalue (index 0) is not shown, as this eigenvalue is equal to one.

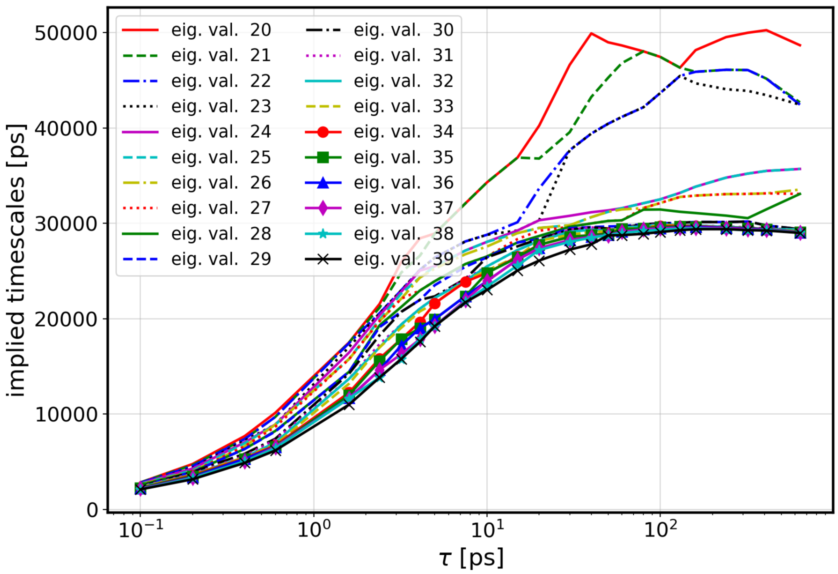

Figure S5: Implied timescales $t_k(\tau) = -\frac{\tau}{\ln \lambda_k(\tau)}$ for $Li_{13}Si_4$ calculated from eigenvalues of $\mathcal{M}^\tau$ for different lag times τ.

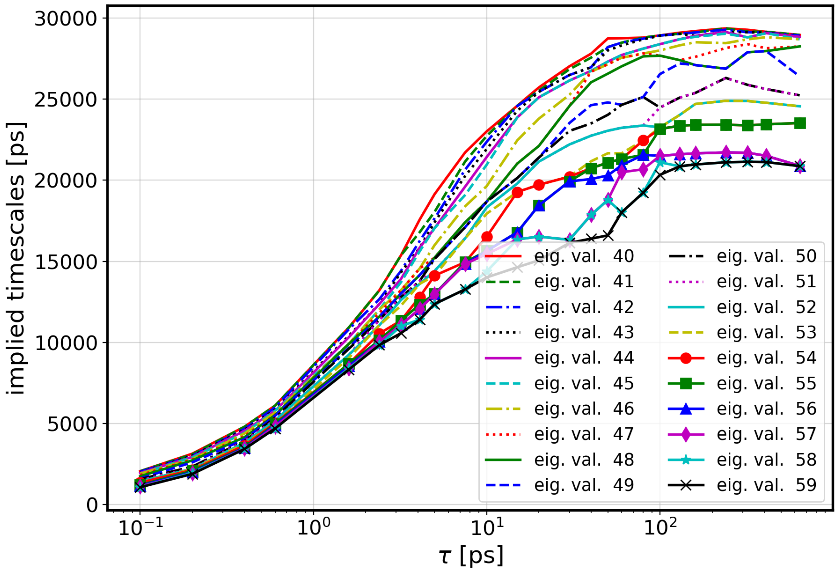

Figure S6: Implied timescales $t_k(\tau) = -\frac{\tau}{\ln \lambda_k(\tau)}$ for $Li_{13}Si_4$ calculated from eigenvalues of $\mathcal{M}^\tau$ for different lag times τ.

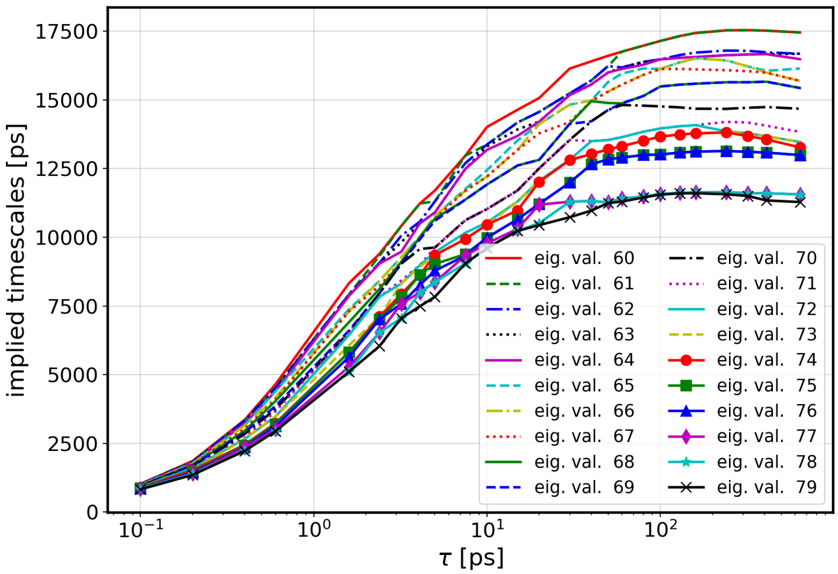

Figure S7: Implied timescales $t_k(\tau) = -\frac{\tau}{\ln \lambda_k(\tau)}$ for $Li_{13}Si_4$ calculated from eigenvalues of $\mathcal{M}^\tau$ for different lag times τ.

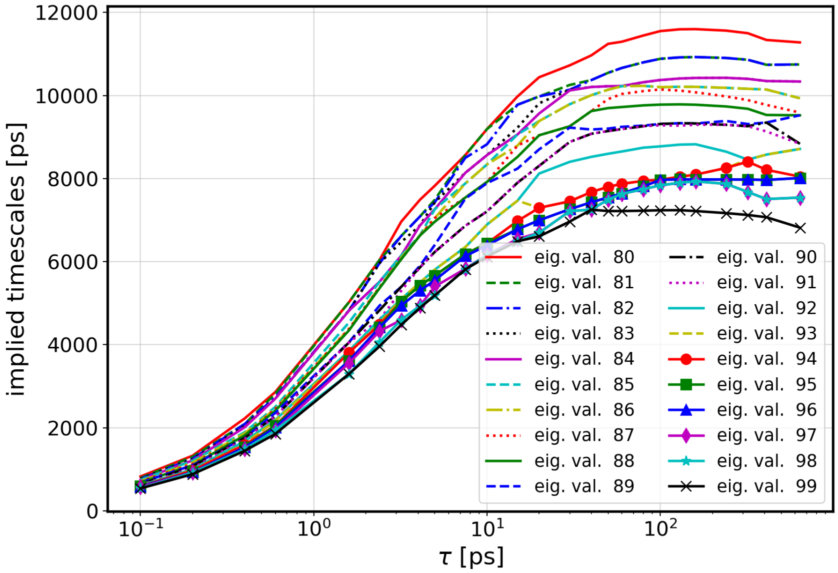

Figure S8: Implied timescales $t_k(\tau) = -\frac{\tau}{\ln \lambda_k(\tau)}$ for $Li_{13}Si_4$ calculated from eigenvalues of $\mathcal{M}^\tau$ for different lag times τ.

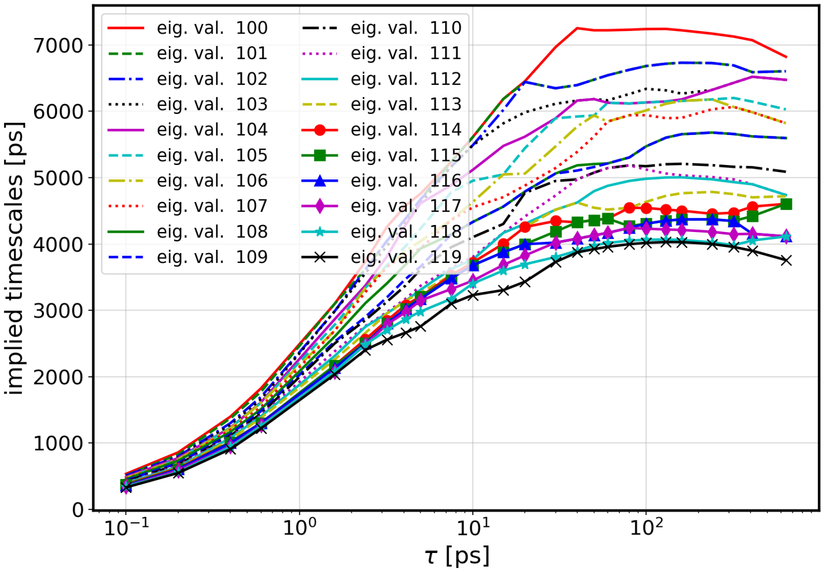

Figure S9: Implied timescales $t_k(\tau) = -\frac{\tau}{\ln \lambda_k(\tau)}$ for $Li_{13}Si_4$ calculated from eigenvalues of $\mathcal{M}^\tau$ for different lag times τ.

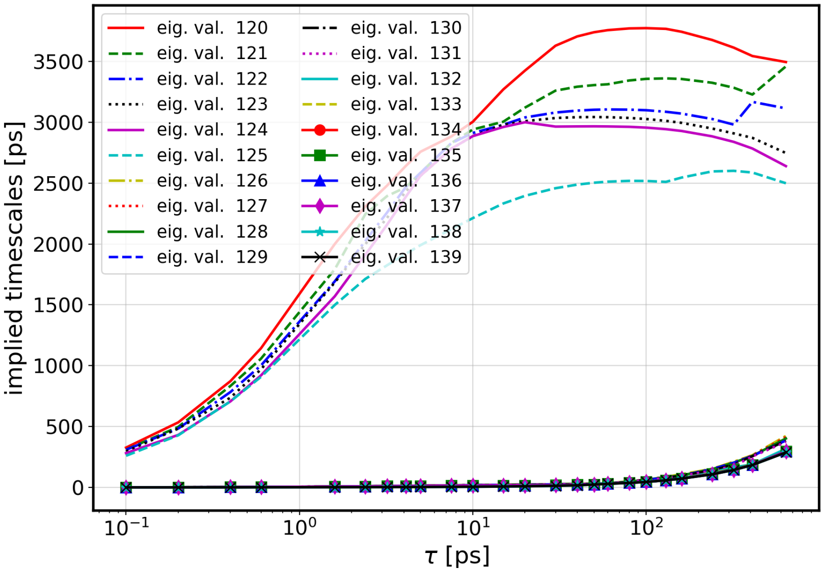

Figure S10: Implied timescales $t_k(\tau) = -\frac{\tau}{\ln \lambda_k(\tau)}$ for $Li_{13}Si_4$ calculated from eigenvalues of $\mathcal{M}^\tau$ for different lag times τ.

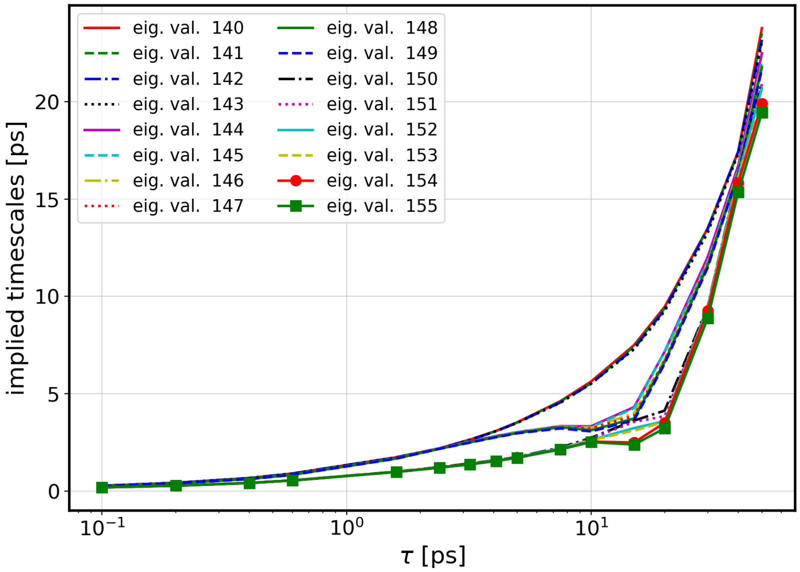

Figure S11: Implied timescales $t_k(\tau) = -\frac{\tau}{\ln \lambda_k(\tau)}$ for $Li_{13}Si_4$ calculated from eigenvalues of $\mathcal{M}^\tau$ for different lag times τ.

1.5 Dimension of the dynamical state space under three idealized ion-transport mechanisms

For ionic transport in general, and in particular for Li_xSi_y, several mechanisms are possible. Ion transport may involve (i) independent ion motion (independent Li jumps), (ii) correlated motion among mobile ions (Li motion correlated with other Li ions), or (iii) correlated motion involving both mobile ions and host atoms (Li motion correlated with Li and Si atoms).

From the instructive toy example from the main part of the manuscript it is clear that the dimension of the state space describing the true dynamics increases drastically from case (i) to case (iii), since correlations involve an increasing number of particles. The number of possible states is determined by all possible distributions of the relevant particles among the available lattice sites. Table S3 summarizes the corresponding state-space sizes and the minimal lag time τ_0 required for the transition matrix to satisfy the CK test. Coarse-graining the dynamics inevitably introduces memory effects and therefore requires a finite lag time τ_0 before the model becomes Markovian. The corresponding minimal lag times are summarized

in Table S3.

Table S3: Minimal lag time τ_0 required for transition matrices constructed on different discretizations of the state space to satisfy the Chapman–Kolmogorov test for Li motion in Li_xSi_y. N denotes the number of Li ions and $\tilde{N}$ the number of available Li lattice sites. M denotes the number of Si atoms and $\tilde{M}$ the number of available Si lattice sites. Green fields indicate that the Markov property is satisfied without lag time ($\tau_0 = 0$), orange fields require a finite lag time ($\tau_0 > 0$), and red fields indicate very large lag times ($\tau_0 \gg 0$). Coarse-graining the dynamics inevitably introduces memory effects and therefore requires a finite lag time τ_0 before the model becomes Markovian.

**Diffusion mechanism**	**Number of discrete states used for MSM**		
	N	$\binom{\tilde{N}}{N}$	$\binom{\tilde{N}}{N}\binom{\tilde{M}}{M}$
Independent Li motion	$\tau_0 = 0$	$\tau_0 = 0$	$\tau_0 = 0$
Li motion correlated with Li ions	$\tau_0 > 0$	$\tau_0 = 0$	$\tau_0 = 0$
Li motion correlated with Li and Si ions	$\tau_0 \gg 0$	$\tau_0 > 0$	$\tau_0 = 0$

1.6 Verification that the transition matrix $\mathcal{M}^{\tau_0}_{\text{sampled}}$ differs from the equilibrium matrix

In this section we verify that the MSMs constructed on the state space defined by the crystallographic lattice sites of the Li atoms are meaningful. For this purpose, we test whether the transition matrix $\mathcal{M}^{\tau_0}_{\text{sampled}}$ differs from the equilibrium matrix $\mathcal{M}^{\text{equil}}$. Here, the equilibrium matrix refers to a matrix whose columns are given by the stationary distribution of the system, and τ_0 denotes the minimal lag time for which the MSM becomes Markovian.

If $\mathcal{M}^{\tau_0}_{\text{sampled}}$ were equal (or very close) to $\mathcal{M}^{\text{equil}}$, the resulting MSM would be trivial, since the system would already be equilibrated on the timescale τ_0 and no meaningful kinetic information could be extracted.

To quantify the difference between these matrices, we evaluate the relative deviation of $\mathcal{M}^{\tau_0}_{\text{sampled}}$ from $\mathcal{M}^{\text{equil}}$ according to

$$\frac{\left\|\mathcal{M}^{\tau_0}_{\text{sampled}} - \mathcal{M}^{\text{equil}}\right\|_2}{\left\|\mathcal{M}^{\tau_0}_{\text{sampled}}\right\|_2}. \tag{1}$$

Figure S12 shows the value of this quantity as a function of the lag time. For lag times

where this deviation is significantly larger than zero, the transition matrix $\mathcal{M}^{\tau_0}_{\mathrm{sampled}}$ clearly differs from the equilibrium matrix, confirming that the MSM retains non-trivial kinetic information.

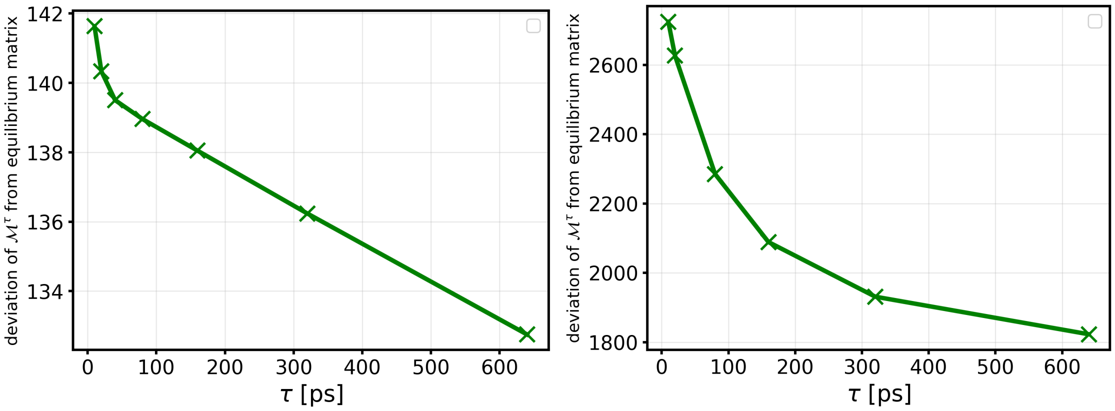

Figure S12: Relative deviation of $\mathcal{M}^{\tau_0}_{\mathrm{sampled}}$ from $\mathcal{M}^{\mathrm{equil}}$ according to eq. 1 for $Li_{13}Si_4$ (left) and $Li_{12}Si_7$ (right).

2 Fine-tuning of foundational Machine learned force fields for amorphous phases

2.1 Computational Details for *ab initio calculations* of amorphous phases

Amorphous Li_xSi structures were generated from force-field molecular dynamics (FFMD) simulations using the LAMMPS program package. The parametrization of a reactive force field (ReaxFF) proposed by Pan *et al.*[S5] was employed as the interatomic potential. Starting from simulation boxes containing random atomic configurations of approximately 400 Li and Si atoms with the desired Li/Si ratio, a melt–quench simulation protocol was applied after initial relaxation of the atomic coordinates to transform these systems into realistic structural models of amorphous Li_xSi. After quenching, geometry optimizations were carried out at the DFT level using a Broyden–Fletcher–Goldfarb–Shanno (BFGS) minimizer to obtain fully

relaxed structures for subsequent AIMD simulations.

Density functional theory (DFT) calculations under periodic boundary conditions were carried out using the CP2K program package with the QUICKSTEP code and the Gaussian plane-wave method. A DZVP-MOLOPT-SR-GTH basis set was employed together with GTH-PBE pseudopotentials. The plane-wave energy cutoff was set to 600 Ry and the relative cutoff to 60 Ry. The PBEsol functional was used to describe the exchange–correlation energy for cell-parameter relaxations, while the PBE functional was applied otherwise. To achieve a self-consistent field convergence threshold of 10^{-6}, Kerker mixing with $\alpha = 0.4$ was applied together with Fermi–Dirac smearing at an electronic temperature of 500 K.

AIMD simulations were performed with a time step of 1 fs in the canonical ensemble (NVT) at 500 K, with temperature control provided by a Nosé–Hoover chain thermostat. The fully relaxed structures were equilibrated for 5 ps using massive thermostatting with a time constant $\tau = 50$ fs, followed by 5 ps with $\tau = 100$ fs, and subsequently by 5 ps with a global thermostat and $\tau = 100$ fs. After equilibration, trajectories of 10 ps length were generated for analysis using global thermostatting with $\tau = 100$ fs.

Climbing-image nudged elastic band (CI-NEB) calculations were performed to map lithium migration paths identified from the AIMD trajectories using either 8 or 16 replicas, including the initial and final structures. Migration paths were detected by fully relaxing snapshots extracted from the AIMD simulations every 100 fs and interpolating atomic coordinates between consecutive snapshots to identify hopping events. A distance-based criterion together with a topological criterion based on neighbor-list changes was used to classify atomic motions as migration paths and to distinguish them from other events such as structural relaxations that frequently occur in amorphous structures.

2.2 Evaluation of fine-tuned MLFFs for amorphous phases

2.3 Force and energy errors for fine-tuned MLFFs for amorphous phases

The fine-tuned MLFFs for the two amorphous phases achieve energy errors below 3 meV atom^{-1} and force errors below 55 meV Å^{-1} for both systems.

Table S4: Validation errors (RMSE) for fine-tuned MACE models on amorphous Li_xSi compositions. Forces and energies were fine-tuned to phase specific data.

Composition	RMSE E [meV/atom]	RMSE F [meV/Å]	relative F RMSE [%]
Li134Si268	0.5	54	10.2
Li300Si100	2.6	35.3	9.53

2.4 Structural and kinetic benchmarks for MLFFs

In line with the benchmarks for the crystalline phases, the accuracy of the MLFFs was also assessed for the amorphous phases against both structural and kinetic benchmarks. Structural fidelity was quantified by computing radial distribution functions (RDFs) $g(r)$ from MLFF trajectories and comparing them with AIMD reference data. RDFs were evaluated for all relevant atomic pairs (Li–Li, Li–Si, and Si–Si), with emphasis on reproducing both peak positions and intensities. Kinetic validation was performed by calculating lithium migration barriers using the climbing-image nudged elastic band (CI-NEB) method. Diffusion pathways were identified from AIMD trajectories, and corresponding initial and final states were optimized with DFT at the PBE level. MLFF-predicted barrier heights were then compared directly against DFT results, providing a stringent test of the models' ability to reproduce the energetics governing lithium transport.

2.4.1 Radial distribution functions for amorphous phases

Figure S13 compares the Radial distribution functions (RDFs) obtained from MLFF molecular dynamics trajectories of $Li_{300}Si_{100}$ and $Li_{134}Si_{268}$ to AIMD reference data at 500 K The fine-tuned MLFF reproduces all Si–Si peak positions and amplitudes.

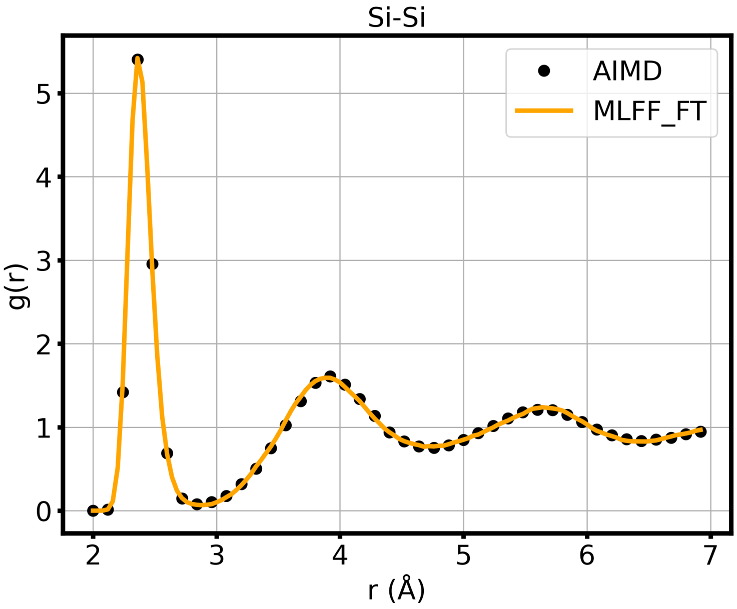

Figure S13: Si-Si radial distribution function g(r) obtained from AIMD (in black filled spheres) and different MACE models for the amorphous phases $Li_{300}Si_{100}$ and $Li_{134}Si_{268}$ computed at 500K.

2.4.2 Nudged elastic band calculations

Figure S14 and Figure S15 illustrates representative several lithium migration pathways identified from AIMD trajectories in both $Li_{300}Si_{100}$ and $Li_{134}Si_{268}$, computed using the nudged elastic band (NEB) method. Across all tested migration paths, the energy profiles predicted by the fine-tuned MLFF show significantly improved agreement with DFT energies compared to those obtained from the pretrained MACE foundation model. The close agreement of barrier heights ensures that lithium jump statistics derived from long MLFF simulations accurately reflect the underlying DFT energy landscape and can be reliably used for transport modeling.

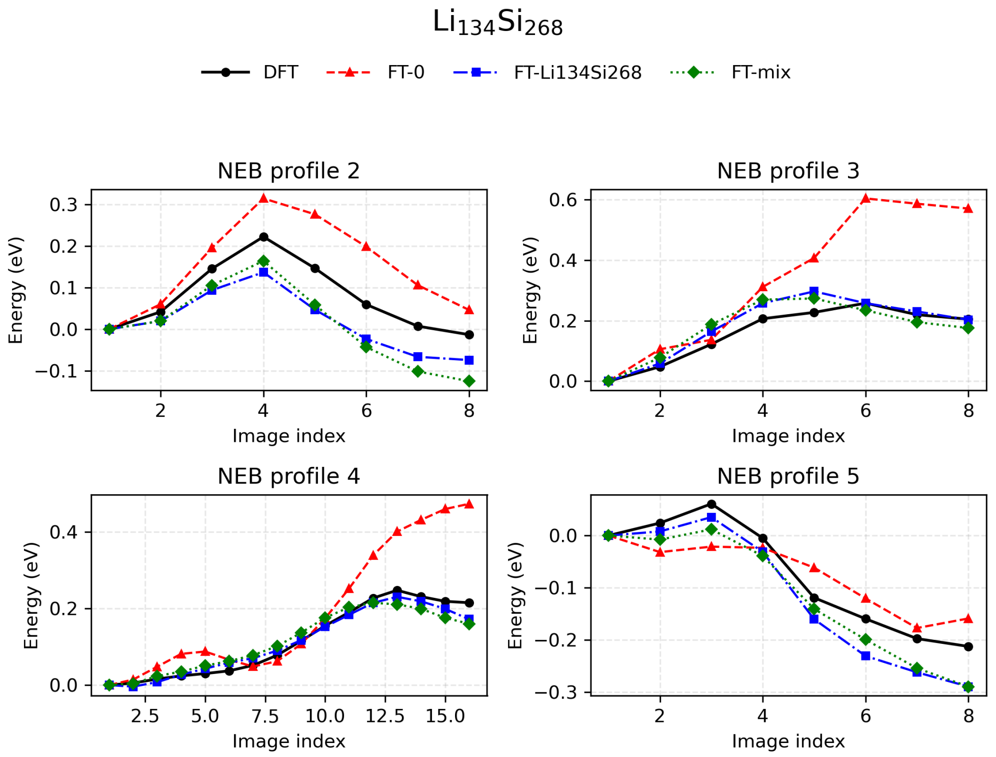

Figure S14: Comparison of several NEB paths of amorphous $Li_{134}Si_{268}$ system, showing that the fine-tuned MLFF accurately reproduces the migration barrier obtained from reference AIMD data, whereas the foundation model either underestimates or overestimates the paths energetics.

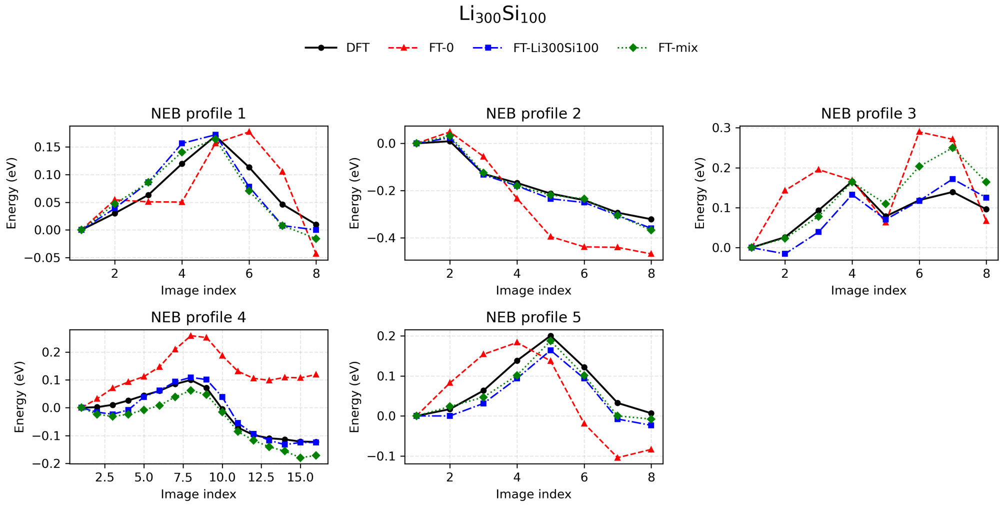

Figure S15: Comparison of several NEB paths of amorphous $Li_{300}Si_{100}$ system, showing that the fine-tuned MLFF accurately reproduces the migration barrier obtained from reference AIMD data, whereas the foundation model either underestimates or overestimates the paths energetics.

References

(S1) Batatia, I.; Kovacs, D. P.; Simm, G.; Ortner, C.; Csányi, G. MACE: Higher order equivariant message passing neural networks for fast and accurate force fields. *Advances in Neural Information Processing Systems* **2022**, *35*, 11423–11436.

(S2) Batatia, I.; Benner, P.; Chiang, Y.; Elena, A. M.; Kovács, D. P.; Riebesell, J.; Advincula, X. R.; Asta, M.; Avaylon, M.; Baldwin, W. J.; Berger, F.; Bernstein, N.; Bhowmik, A.; Bigi, F.; Blau, S. M.; Cărare, V.; Ceriotti, M.; Chong, S.; Darby, J. P.; De, S.; Pia, F. D.; Deringer, V. L.; Elijošius, R.; El-Machachi, Z.; Falcioni, F.; Fako, E.; Ferrari, A. C.; Gardner, J. L. A.; Gawkowski, M. J.; Genreith-Schriever, A.; George, J.; Goodall, R. E. A.; Grandel, J.; Grey, C. P.; Grigorev, P.; Han, S.; Handley, W.; Heenen, H. H.; Hermansson, K.; Holm, C.; Ho, C. H.; Hofmann, S.; Jaafar, J.; Jakob, K. S.; Jung, H.; Kapil, V.; Kaplan, A. D.; Karimitari, N.; Kermode, J. R.; Kourtis, P.; Kroupa, N.; Kullgren, J.; Kuner, M. C.; Kuryla, D.; Liepuoniute, G.; Lin, C.; Margraf, J. T.; Magdău, I.-B.; Michaelides, A.; Moore, J. H.; Naik, A. A.; Niblett, S. P.; Norwood, S. W.; O'Neill, N.; Ortner, C.; Persson, K. A.; Reuter, K.; Rosen, A. S.; Rosset, L. A. M.; Schaaf, L. L.; Schran, C.; Shi, B. X.; Sivonxay, E.; Stenczel, T. K.; Svahn, V.; Sutton, C.; Swinburne, T. D.; Tilly, J.; van der Oord, C.; Vargas, S.; Varga-Umbrich, E.; Vegge, T.; Vondrák, M.; Wang, Y.; Witt, W. C.; Wolf, T.; Zills, F.; Csányi, G. A foundation model for atomistic materials chemistry. 2025; `https://arxiv.org/abs/2401.00096`.

(S3) Deng, B.; Zhong, P.; Jun, K.; Riebesell, J.; Han, K.; Bartel, C. J.; Ceder, G. CHGNet as a pretrained universal neural network potential for charge-informed atomistic modelling. *Nature Machine Intelligence* **2023**, *5*, 1031–1041.

(S4) Hänseroth, J.; Flötotto, A.; Qaisrani, M. N.; Dreßler, C. Fine-Tuning Unifies Foundational Machine-learned Interatomic Potential Architectures at ab initio Accuracy. *arXiv preprint arXiv:2511.05337* **2025**,

(S5) Pan, L.-Y.; Kuo, C.-L. Atomistic study on the origins of the anisotropic lithiation behaviors of the silicon anode using the reactive force field based molecular dynamics simulations. *Acta Materialia* **2024**, *265*, 119610.
